# The X‐linked Becker muscular dystrophy (*bmx*) mouse models Becker muscular dystrophy via deletion of murine dystrophin exons 45–47

**DOI:** 10.1002/jcsm.13171

**Published:** 2023-01-11

**Authors:** Christopher R. Heier, Nikki M. McCormack, Christopher B. Tully, James S. Novak, Breanne L. Newell‐Stamper, Alan J. Russell, Alyson A. Fiorillo

**Affiliations:** ^1^ Center for Genetic Medicine Research Children's National Hospital Washington DC USA; ^2^ Department of Genomics and Precision Medicine George Washington University School of Medicine and Health Sciences Washington DC USA; ^3^ Edgewise Therapeutics, BioFrontiers Institute University of Colorado Boulder CO 80303 USA

**Keywords:** Becker muscular dystrophy, dystrophin, exon skipping, Duchenne muscular dystrophy, inflammation, microRNAs, dystrophin‐associated proteins

## Abstract

**Background:**

Becker muscular dystrophy (BMD) is a genetic neuromuscular disease of growing importance caused by in‐frame, partial loss‐of‐function mutations in the dystrophin (*DMD*) gene. BMD presents with reduced severity compared with Duchenne muscular dystrophy (DMD), the allelic disorder of complete dystrophin deficiency. Significant therapeutic advancements have been made in DMD, including four FDA‐approved drugs. BMD, however, is understudied and underserved—there are no drugs and few clinical trials. Discordance in therapeutic efforts is due in part to lack of a BMD mouse model which would enable greater understanding of disease and de‐risk potential therapeutics before first‐in‐human trials. Importantly, a BMD mouse model is becoming increasingly critical as emerging DMD dystrophin restoration therapies aim to convert a DMD genotype into a BMD phenotype.

**Methods:**

We use CRISPR/Cas9 technology to generate *bmx* (*B*ecker *m*uscular dystrophy, *X*‐linked) mice, which express an in‐frame ~40 000 bp deletion of exons 45–47 in the murine *Dmd* gene, reproducing the most common BMD patient mutation. Here, we characterize muscle pathogenesis using molecular and histological techniques and then test skeletal muscle and cardiac function using muscle function assays and echocardiography.

**Results:**

Overall, *bmx* mice present with significant muscle weakness and heart dysfunction versus wild‐type (WT) mice, despite a substantial improvement in pathology over dystrophin‐null *mdx52* mice. *bmx* mice show impaired motor function in grip strength (−39%, *P* < 0.0001), wire hang (*P* = 0.0025), and *in vivo* as well as *ex vivo* force assays. In aged *bmx*, echocardiography reveals decreased heart function through reduced fractional shortening (−25%, *P* = 0.0036). Additionally, muscle‐specific serum CK is increased >60‐fold (*P* < 0.0001), indicating increased muscle damage. Histologically, *bmx* muscles display increased myofibre size variability (minimal Feret's diameter: *P* = 0.0017) and centrally located nuclei indicating degeneration/regeneration (*P* < 0.0001). *bmx* muscles also display dystrophic pathology; however, levels of the following parameters are moderate in comparison with *mdx52*: inflammatory/necrotic foci (*P* < 0.0001), collagen deposition (+1.4‐fold, *P* = 0.0217), and sarcolemmal damage measured by intracellular IgM (*P* = 0.0878). Like BMD patients, *bmx* muscles show reduced dystrophin protein levels (~20–50% of WT), whereas *Dmd* transcript levels are unchanged. At the molecular level, *bmx* muscles express increased levels of inflammatory genes, inflammatory miRNAs and fibrosis genes.

**Conclusions:**

The *bmx* mouse recapitulates BMD disease phenotypes with histological, molecular and functional deficits. Importantly, it can inform both BMD pathology and DMD dystrophin restoration therapies. This novel model will enable further characterization of BMD disease progression, identification of biomarkers, identification of therapeutic targets and new preclinical drug studies aimed at developing therapies for BMD patients.

## Introduction

Becker muscular dystrophy (BMD) is a debilitating X‐linked muscle disease caused by in‐frame mutations of the dystrophin gene.^1^, ^2^, ^3^ BMD‐causing mutations result in production of a truncated isoform of dystrophin protein that is partially functional and expressed at reduced amounts. Patients have a variable presentation; some individuals show severe muscle weakness in early childhood with loss of ambulation by late teens to early 20s, whereas others remain largely asymptomatic.^4^, ^5^, ^6^, ^7^, ^8^ Ultimately, heart problems develop in most patients, with up to 50% of BMD patients eventually dying from cardiomyopathy.^9^, ^10^ As a rare disease with variable pathology and no current animal model, BMD represents an understudied and underserved group with no approved therapy and very few clinical trials (two interventions in active clinical trials for BMD vs. 30 for DMD). Development of clinically pertinent BMD mouse models will facilitate an improved understanding of disease pathology and functionality of dystrophin isoform domains while also providing a preclinical platform for development of the first BMD therapeutics.

BMD patients show variability in dystrophin protein levels that partially correlate with disease severity, though even ‘mild’ patients exhibit muscle pathology.^2^, ^11^, ^12^, ^13^, ^14^, ^15^ Inter‐patient and intra‐patient variability is observed with respect to dystrophin protein amount. Specifically, variable and reduced dystrophin protein levels are observed in muscle biopsies from different patients, from different muscles and even within different regions of a single muscle biopsy. Elevated serum CK levels are present at birth, and up to 95% of patients can be detected by screening for serum CK levels. Muscle biopsies show increases in fibre size variability, centralized nuclei, fibre degeneration and fibre branching.^4^, ^6^, ^7^ Muscle atrophy and pseudohypertrophy are also seen, with fibrosis or fatty replacement of muscle.^16^

BMD provides hope to the Duchenne muscular dystrophy (DMD) community because it provides evidence that expressing a truncated dystrophin isoform can result in a milder disease. DMD is caused by a complete loss of functional dystrophin and is more severe with an earlier onset. A promising area of therapeutics seeks to restore expression of ‘Becker‐like’ isoforms of dystrophin through mRNA‐splice modulating antisense oligos (AOs). Preclinical dystrophinopathy research has focused primarily on DMD because there are several available mouse models that recreate dystrophin‐null mutations. These include *mdx23*, *mdx52* and several CRISPR mouse and rat models.^17^, ^18^, ^19^, ^20^, ^21^ Importantly, studies in *mdx* mice have led to development and accelerated approval of four exon‐skipping AO drugs for DMD (eteplirsen, golodirsen, viltolarsen and casimersen). Although these dystrophin restoration therapies are promising, they are not curative. In the best‐case scenario, these therapeutics would convert a DMD genotype into a milder BMD phenotype. BMD therefore serves as a model for efficient dystrophin restoration therapies, and as these therapies enter widespread clinical use, there will be a further need for development of BMD therapeutics^9^ and disease models.

Here we introduce a large (approximately 40 000 bp) deletion mutation via CRISPR to remove exons 45–47 of the endogenous murine dystrophin (*Dmd*) gene, recreating the most common BMD‐causing deletion in humans. The resulting *bmx* mouse model (for *B*ecker *m*uscular dystrophy, *X*‐linked) has molecular, histopathological and functional deficits consistent with BMD patients while displaying phenotypes that are intermediate to control wild‐type (WT) and DMD model (*mdx52*) mice. These studies establish the *bmx* mouse as a novel model of BMD. Moving forward, the *bmx* mouse can be used to gain new insights into BMD disease pathology, to model dystrophin restoration therapies in DMD and to facilitate the development of BMD therapeutics as well as DMD co‐therapeutics.

## Methods

### Mice

All animal studies were conducted according to the NIH Guide for the Care and Use of Laboratory Animals, all national laws and 1964 Declaration of Helsinki standards and amendments and approval of the Institutional Animal Care and Use Committee of Children's National Hospital (CNH). *C57/BL6‐mdx∆52* mice (*mdx52*) contain a deletion of exon 52 of the *Dmd* gene, resulting in absence of full‐length dystrophin,^21^ and were originally provided as a gift from Dr Shin'ichi Takeda. Wild‐type C57/BL6 (WT) mice were purchased from Jackson Laboratory (Bar Harbor, ME). All strains are currently maintained in‐house at CNH.

### Generation of bmx mice

*bmd∆45‐47* (*bmx*) mice were generated on a C57/BL6 background via CRISPR using guide RNAs (gRNAs) to delete exons 45–47 of the murine dystrophin (*Dmd*) gene. gRNAs were designed to target protospacer adjacent motif (PAM) sequences upstream of *Dmd* exon 45 and downstream of *Dmd* exon 47. Subsequent DNA sequencing of pups was performed to verify germline transmission.

### Serum creatine kinase

Serum creatine kinase, muscle‐type (CKM) was assayed via ELISA according to manufacturer's instructions (Novus Biologicals #NBP2‐75306). Blood collection and serum isolation details can be found in the *Supporting Information*.

### Capillary western immunoassay (Wes)

Muscles were flash frozen in liquid‐nitrogen. Eight‐micrometre sections were lysed in High SDS buffer containing 0.02% EDTA (pH 8.0), 0.075% Tris–HCL (pH 6.8) and protease inhibitors. Capillary Western immunoassay (Wes) was performed according to manufacturer's instructions using 66–440 kDa Separation Modules (ProteinSimple). Details can be found in the *Supporting Information*.

### Motor function tests

*Grip strength*: Forelimb and hindlimb grip strength was assessed using a grip strength meter (Columbus Instruments) daily for 2 consecutive days according to Treat NMD protocols (DMD_M.2.2.001), with data interpreted as averaged maximum daily values. *Wire and box hang*: Two‐limb wire hang and four‐limb grid hang tests were performed in accordance with Treat NMD protocols (DMD_M.2.1.005). Details can be found in the *Supporting Information*.

### In vivo isometric torque

To measure *in vivo* torque production of the anterior crural *muscles* [TA, extensor digitorum longus (EDL), peroneus tertius and extensor hallucis longus], mice were anesthetized with 1.5% isoflurane‐mixed O_2_ and hair removed from lower hindlimbs, whereas the foot was attached to the dual‐mode lever and maintained at 90° for isometric torque assessment (Aurora Scientific). See *Supporting Information* for full details.

### Ex vivo eccentric contractions

An eccentric injury protocol was performed in male *bmx, mdx52* and WT mice based on previously reported protocols. Details and citations can be found in the *Supporting Information*.

### Echocardiography

See *Supporting Information* for full details and citations.

### Immunofluorescence

Muscles were mounted, frozen in liquid‐nitrogen cooled isopentane and sectioned (8 μm) onto slides. For most immunofluorescence, muscle sections were fixed in ice‐cold acetone for 10 min. For anti‐IgM immunofluorescence, muscle sections were fixed with 4% paraformaldehyde for 10 min at room temperature. Slides were washed, blocked for 1 h (1X PBST with 0.1% Triton X‐100, 1% BSA, 10% goat serum and 10% horse serum), washed three times, then exposed to primary antibodies overnight at 4°C and secondary antibodies for 1 h at RT. Coverslips were mounted using Prolong Gold Mounting Medium with DAPI. Slides were imaged using an Olympus VS‐120 scanning microscope at 20×. Antibody dilutions can be found in the *Supporting Information*.

### BrdU staining

Mice aged 8–10 weeks were administered water containing 0.8 mg/mL BrdU for 1 week followed by normal water for 1 week. Muscle was then stained for BrdU. Full details can be found in the *Supporting Information*.

### Gene expression

qRT‐PCR of miRNAs and mRNAs was performed. Details and assay IDs are provided in the *Supporting Information*.

## Results

### Generation of a Becker muscular dystrophy mouse model

**Figure 1:**
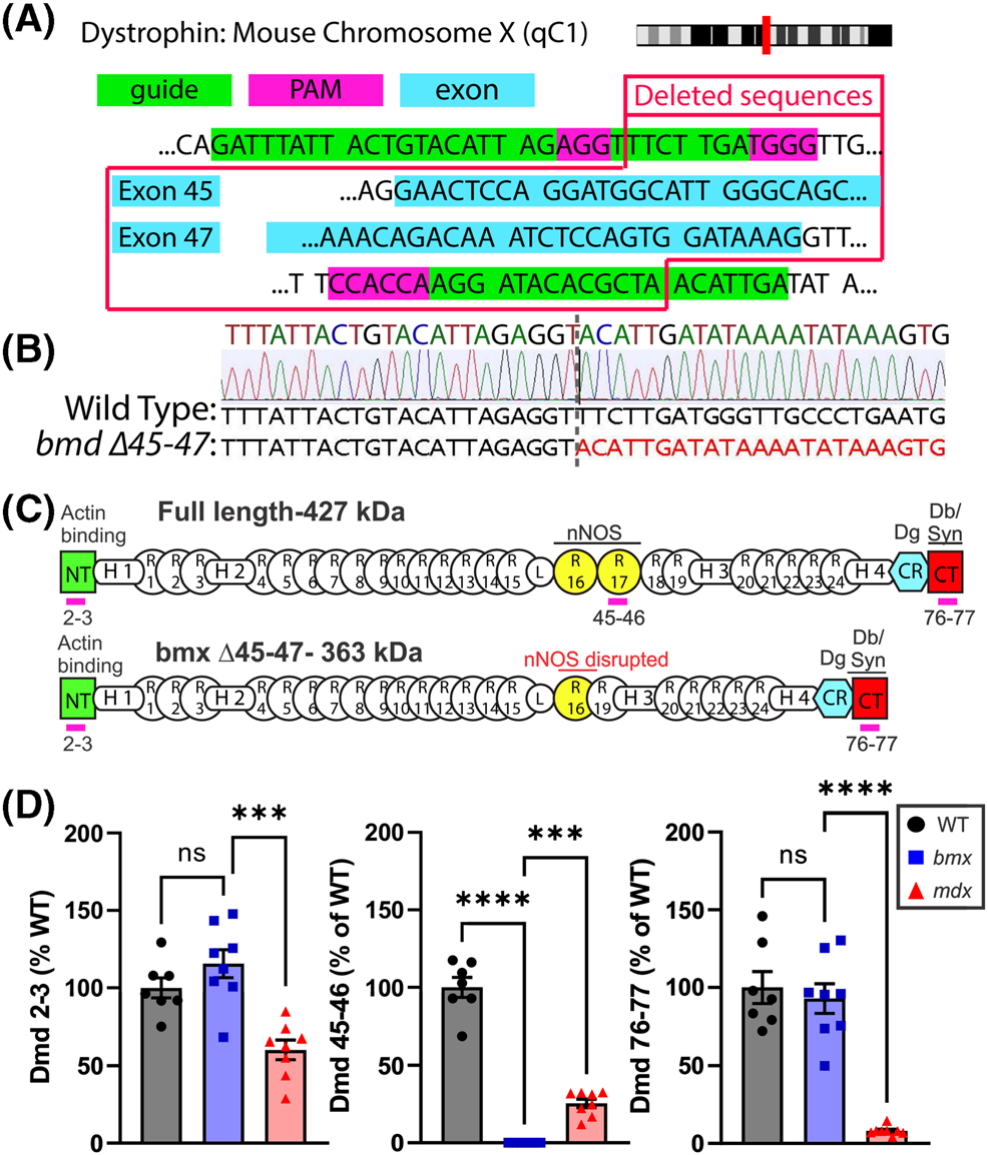
Generation of the *bmx* mouse model of BMD by deletion of dystrophin exons 45–47. (A) Schematic of the dystrophin gene showing CRISPR/Cas9‐targeted deletion of exons 45–47. (B) DNA sequencing showing genomic deletion of dystrophin exons 45–47. (C) Protein structure of dystrophin Δ45–47 shows disruption of the nNOS binding domain and a disruption of the rod domain that results in an out‐of‐phase spectrin‐type repeat (STR) pattern. (D) mRNA levels of dystrophin exons 2–3 and exons 76–77 are unchanged in Becker muscular dystrophy (*bmx*) mice, whereas dystrophin exons 45–46 are deleted in quadriceps muscle. NT = amino‐terminus, H = Hinge, R = STR, CR = cysteine‐rich, CT = carboxy‐terminus. ANOVA; *n* = 7–8; ***P < 0.001, ****P ≤ 0.0001.

To create *bmx* mice (for *B*ecker *m*uscular dystrophy, *X*‐linked) that model the most common BMD patient mutation,^14^ we used CRISPR/Cas9 to introduce a ~40 000 bp genomic deletion into the endogenous murine dystrophin (*Dmd*) gene, excising exons 45–47. Guide RNAs (gRNAs) were designed to target protospacer adjacent motif (PAM) sequences upstream of *Dmd* exon 45 and downstream of *Dmd* exon 47 (*Figure* 1A). DNA sequencing confirmed genomic deletion of dystrophin exons 45–47 (*Figure* 1B), which is predicted to disrupt spectrin‐type repeats (STRs) and neuronal nitric oxide synthase (nNOS) binding (*Figure* 1C). qRT‐PCR was used to validate deletion of exons 45–47 and to quantify overall *Dmd* transcript levels. We observed similar expression of *Dmd* between WT and *bmx* mice using probes at the 5′ and 3′ ends of the *Dmd* transcript (exon 2–3 or 76–77) while using a probe for exons *Dmd* 45–46 confirmed *bmx* quadriceps (*Figure* 1D), TA, heart and diaphragm (*Figure* S1) lack this region. We observed progressively reduced amounts of *Dmd* mRNA in the 5′–3′ direction in *mdx52* consistent with 3′ destabilization as previously reported (*Figure* 1D).^22^

### bmx mice present with impaired motor function and reduced muscle force

**Figure 2:**
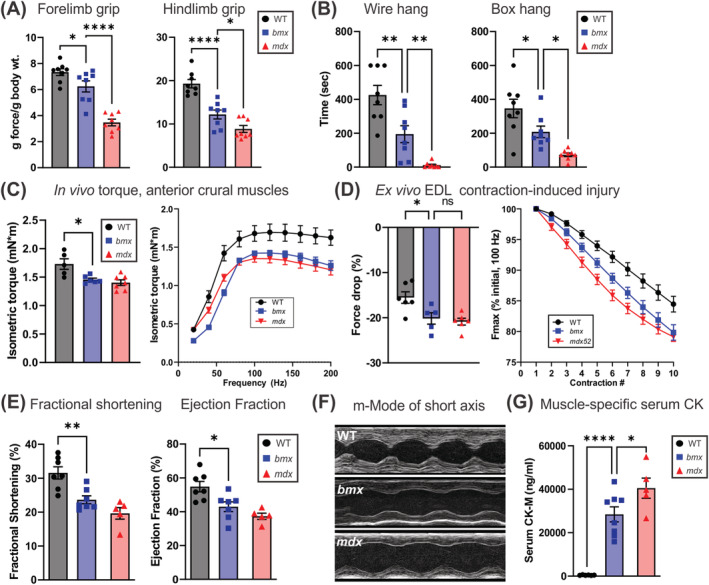
The *bmx* mouse has reduced motor function, muscle force and heart function. (A) Grip strength of *bmx* mice was reduced in both the forelimb (*P* = 0.0465) and hindlimb (*P* = 0.0002) grip strength tests. *n* = 8. (B) Suspension time of *bmx* mice was reduced in the wire hang test (*P* = 0.0087) and in the box hang test (*P* = 0.0489). *n* = 8. (C) *In vivo* maximum isometric torque and torque‐frequency curve for anterior crural muscles of WT, *bmx*, and *mdx52* mice. Maximum isometric torque was reduced in *bmx* mice (*P* = 0.0110). *n* = 6–7. (D) Left; *ex vivo* eccentric contraction‐induced lengthening force drop in EDL, *bmx* shows increased injury (force drop) versus WT. (*P* = 0.0249); right; force drop, expressed as a percent of initial eccentric contraction force, is shown across 10 eccentric contractions. (E) Echocardiography of aged (18‐month‐old) *bmx* mice shows a deficit in heart function through a decrease in fractional shortening (*P* = 0.0036) and ejection fraction (*P* = 0.0131). *n* = 5–7. (F) Representative M‐mode images of the parasternal short axis are provided. (G) Serum CKM levels in aged (1‐year‐old) mice assayed via ELISA. *n* = 6–7. ANOVA, *P ≤ 0.05, **P ≤ 0.01, ***P ≤ 0.001, ****P ≤ 0.0001.

To determine whether *bmx* mice exhibit functional impairments, we performed phenotyping at 10 weeks (forelimb/hindlimb grip strength), 14 weeks (two‐limb wire hang), and 15 weeks (four‐limb grid hang). *bmx* mice showed reduced grip strength versus WT for forelimb (14.90% decrease, *P* = 0.0465) and hindlimb (36.81% decrease, *P <* 0.0001) (*Figure* 2A). Reduced suspension times for *bmx* were also found in wire hang (−54.25%, *P* = 0.0087) and box hang (−39.85%, *P* = 0.0489) (*Figure* 2B). Assaying *in vivo* isometric torque produced in the tibialis anterior (TA), *bmx* mice showed significantly reduced maximum isometric torque vs. WT mice (1.465 vs. 1.650 mN*m, *P* = 0.0110), though there was no significant improvement in *bmx* versus *mdx52* (*Figure* 2C, left). Additionally, the deficit in isometric torque was observed at all frequencies for *bmx* (*Figure* 2C, right). *In vivo* specific isometric torque showed modest reductions in maximal values for *bmx* versus WT (48.74 vs. 53.63 mN*m/kg, *P* = 0.1235) and were significantly elevated versus *mdx52* (*P* = 0.0295) (*Figure* S2A).

We next sought to examine contractile performance and resistance to eccentric injury in *bmx* mice. Ten eccentric contractions of WT EDL muscle resulted in a 15.53% drop in *ex vivo* peak force. In contrast, this protocol reduced peak force by 20.16% in *bmx* EDL muscles (*P* = 0.0239 *bmx* vs. WT) and 20.86% in *mdx* EDL (*Figure* 2D). This injury protocol also resulted in a 42.49% drop in isometric force in *bmx* EDL (*P* = 0.0307) vs. 33% isometric force drop in WT and a 38% drop in *mdx* EDL (*Figure* S2B).

### Aged bmx mice develop heart dysfunction and increased serum CK

Heart function was assessed via echocardiography in 18‐month‐old WT, *bmx* and *mdx52* mice. We chose this age because reduced heart function is observed in *mdx52* mice starting around 12 months of age and reasoned that comparable heart function declines in *bmx* should therefore be present by 12–18 months of age. *bmx* mice showed significant declines in heart function measures (*Figure* 2E,F) including fractional shortening and ejection fraction (−25.07%, *P* < 0.0036, −27.67% *P* < 0.0131 vs. WT). We also assayed serum creatine kinase levels in aged mice; *bmx* mice showed an ~62‐fold (*P* < 0.0001) increase in serum creatine kinase, muscle‐type (CKM) over WT (Figure 2G). *bmx* serum CKM levels (28 447 ng/ml) were intermediate to WT (457 ng/ml) and *mdx* (40 514 ng/ml), and *bmx* CKM was significantly lower than *mdx* (*P* = 0.0217). These data indicate aged *bmx* mice have reduced heart function and increased serum CK, consistent with human disease.

### Pathology of bmx mouse muscle

**Figure 3:**
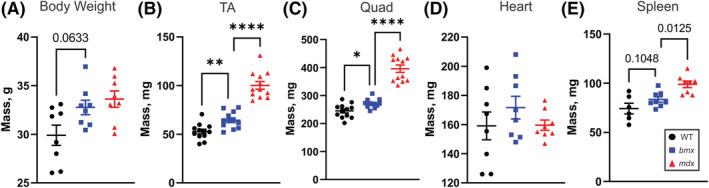
*bmx* mice have increased muscle mass. Body and tissue weights of age‐matched WT, *bmx* and *mdx52* mice were assayed at 5 months of age. (A) *bmx* mice show moderate increases in body mass at 5 months (*P* = 0.0633; n = 8). (B,C) The weight of the tibialis anterior (TA; *P* = 0.0096) and (C) quadriceps (*P* = 0.0456; *n* = 12) is significantly increased in *bmx* mice. (D) Heart weight in *bmx* mice is similar to that of WT mice (*P* = 0.4288; *n* = 8). (E) Spleen weight was elevated in *bmx* mice but was not significant (*P* = 0.1048; *n* = 6–8). ANOVA, *P ≤ 0.05, **P ≤ 0.01, ****P < 0.0001.

In *mdx* mice, limb muscles undergo dramatic necrosis, which is followed by regeneration, increased hypertrophy and increased muscle mass.^23^ Loss of muscle mass then occurs in older *mdx* mice (12–18 months).^23^, ^24^ BMD patients also have both muscle pseudohypertrophy and true hypertrophy.^6^, ^25^ Examining body and muscle mass at 5 months, *bmx* showed a 9.57% increase (*P* = 0.0633) in body weight compared with WT mice (*Figure* 3A). Increased muscle mass was present in every skeletal muscle examined: TA (18.16% increase, *P* = 0.0096), quadriceps (9.75% increase, *P* = 0.0456), gastrocnemius (9.135% increase, *P* = 0.0143), and triceps (17.95% increase, *P* = 0.0583) (*Figures* 3B,C and S3). No significant difference was seen in heart mass (*P* = 0.4288) versus WT and *bmx* mice (*Figure* 3D). As *mdx52* spleens are enlarged due to increased systemic inflammation, we also examined spleen mass. We observed a moderate increase in *bmx* spleen mass, which did not reach significance (12.45% increase, *P* = 0.1048) (*Figure* 3E), but may suggest increased circulating inflammation in *bmx*.

**Figure 4:**
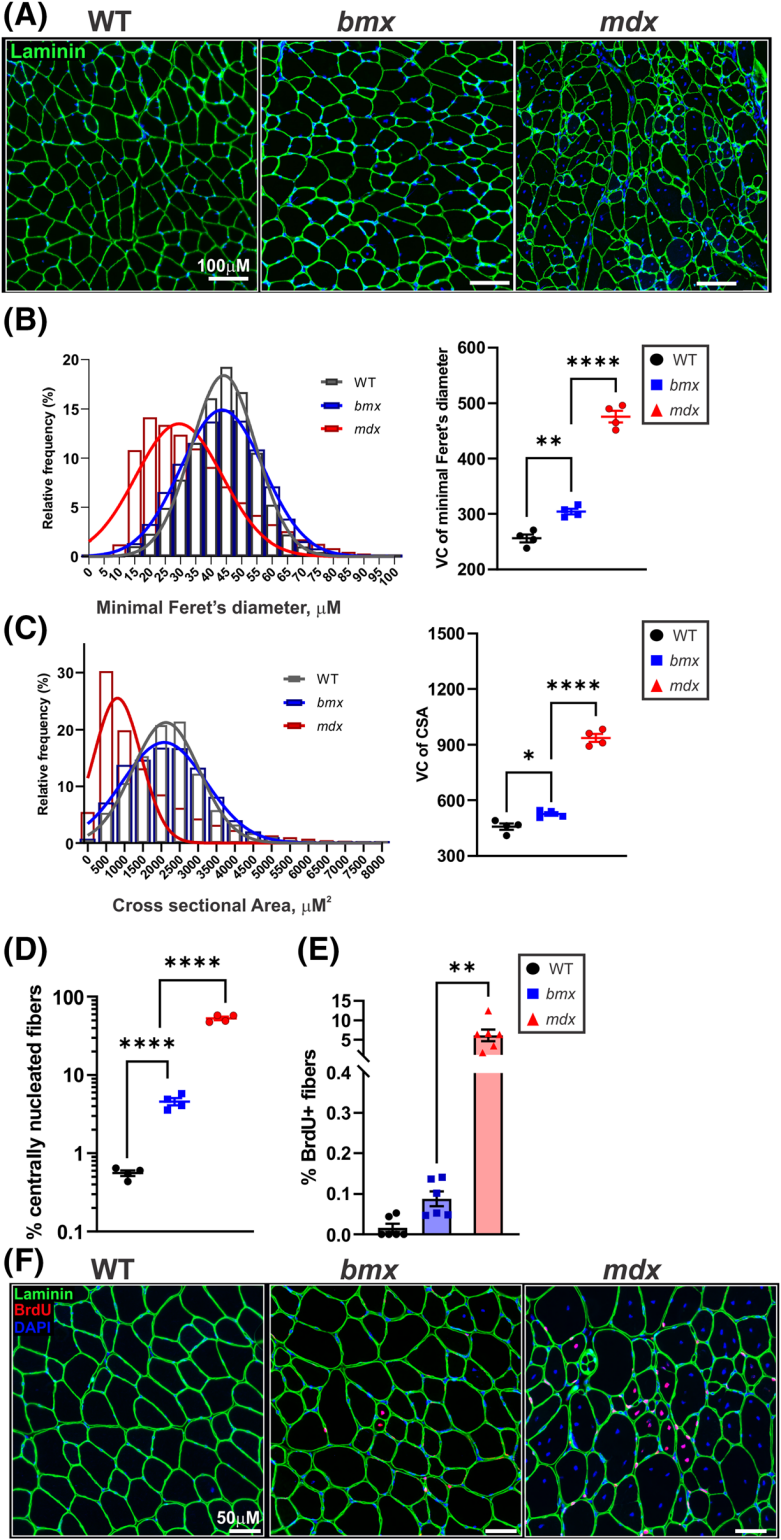
Muscles from *bmx* mice show centrally localized nuclei and increased variation in fibre size. (A) Laminin immunofluorescence of WT, *bmx* and *mdx52* gastrocnemius muscle. DAPI was used as counterstain to visualize myonuclei. Bar = 100 μM. (B) Histogram of minimal Feret's diameter and the variance coefficient (VC) of minimal Feret's diameter. *Bmx* myofibres have increased variation of minimal Feret's diameter (*P* = 0.0017; *n* = 4). (C) Histogram of myofibre cross sectional area (CSA) and VC of myofibre CSA. *bmx* myofibres have increased variation of CSA (*P* = 0.0182; *n* = 4). (D) The percentage of centrally nucleated myofibres was increased in *bmx* mice (*P* < 0.0001; *n* = 4). (e) % of BrdU+ fibres in the tibialis anterior (*P* = 0.0058; *n* = 6). (F) BrdU immunofluorescence, Bar = 50 μM. ANOVA, *P ≤ 0.05, **P ≤ 0.01, ***P ≤ 0.001.

BMD muscle biopsies show increased myofibre size variability and centrally nucleated fibres, indicative of increased and asynchronous muscle regeneration.^6^, ^26^ To examine *bmx* muscle architecture, gastrocnemius muscle sections were immunostained for laminin‐α2. Visually, we observed fibre size variability and some centrally localized nuclei in *bmx* muscles, indicating pathology (*Figure* 4A). Muscle fibre measurements including minimal Feret's diameter and myofibre cross‐sectional area (CSA) were then determined.^27^ Whereas *mdx52* showed a shift towards smaller, regenerating fibres, with some intermittent large fibres, *bmx* mice showed a greater number of both smaller and larger myofibres versus WT, as indicated by histograms plotting minimal Feret's diameter and CSA measurements (*Figure* 4B,C). Differences in fibre size variation were additionally determined by calculating variance coefficients (VCs). VCs showed significant differences between fibre size variability for *bmx* versus WT, for minimal Feret's diameter (+18.91%, *P* = 0.0017) and CSA (+14.96%, *P* = 0.0182) (*Figure* 4B,C). *bmx* also had significantly more centrally nucleated fibres versus WT (4.573% vs. 0.5594%, *P* = 0.0002) (*Figure* 4D).

We next used the thymidine analogue bromodeoxyuridine (BrdU) to label recently generated myofibres, as BrdU incorporates into newly synthesized DNA and therefore labels newly ‘born’ myofibres. Mice were given BrdU for 7 days at 19 weeks of age; BrdU+ centralized nuclei‐containing myofibres were counted as an indicator of myoblasts that had proliferated and fused into myofibres during labelling. Approximately five per cent of *mdx52* myofibres were BrdU+ after 1 week of labelling **(** *Figure* 4E,F). Although there were very few BrdU+ fibres in WT TAs, *bmx* showed a significant increase in the percentage of actively regenerating muscle fibres, which was intermediate to WT and *mdx52* (0.1%, *P* = 0.0058; *Figure* 4E,F). These data show that *bmx* mice have increased regeneration and myofibre hypertrophy versus WT muscles while also having significantly less severe pathology versus *mdx52*.

### bmx mice express reduced dystrophin protein levels

**Figure 5:**
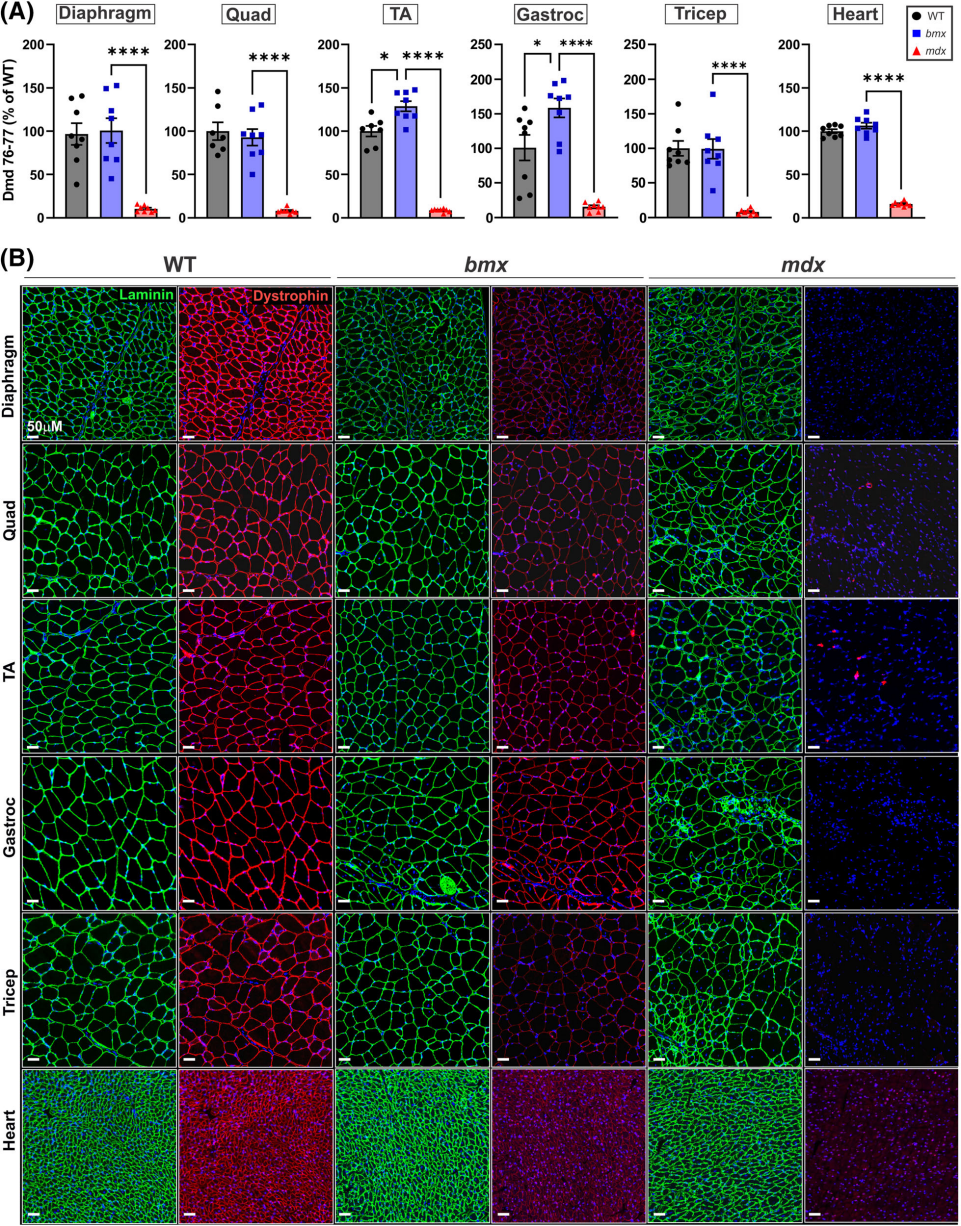
Dystrophin protein levels, but not RNA, are reduced in *bmx* mice. (A) qRT‐PCR showing levels of *Dmd* mRNA as measured by a probe specific to the exon 76–77 junction in the diaphragm, quadriceps, tibialis anterior, gastrocnemius, triceps and heart of WT, *bmx* and *mdx52* mice. *n* = 7–8. (B) Dystrophin (red) and laminin (green) immunofluorescence staining shows reduction of dystrophin in skeletal and cardiac muscles in *bmx* mice. DAPI was used as a counterstain to visualize myonuclei. Bar = 50 μM. ANOVA, *P ≤ 0.05, ****P 0.0001.

We previously reported BMD patients with deletion of *DMD* exons 45–47 have decreased and variable dystrophin protein levels, whereas mRNA levels are unchanged.^15^ To determine if *bmx* mice recapitulate this phenotype, we first performed qRT‐PCR using a probe against the *Dmd* 76–77 exon junction. We chose this junction as previous work shows that a 3–5′ destabilization of *Dmd* mRNA occurs in *mdx52* and DMD muscle, so we reasoned a 3′‐specific probe was more likely to show apparent differences in transcript abundance and stability.^22^ In all muscles measured (diaphragm, quad, TA, gastroc, triceps, heart), *Dmd* transcript levels were not decreased in *bmx*, whereas expectedly, *Dmd* was reduced in *mdx52* muscles (*Figure* 5A). Additionally, in both TA and gastrocnemius muscles, *bmx* showed higher levels of *Dmd* mRNA than WT (TA + 28.85%, *P* = 0.0005, gastroc +57.54%, *P* = 0.0061). This shows that there is not a *Dmd* gene expression deficit in *bmx*, and conversely, in some muscles, *Dmd* transcript levels are more abundant than in WT.

**Figure 6:**
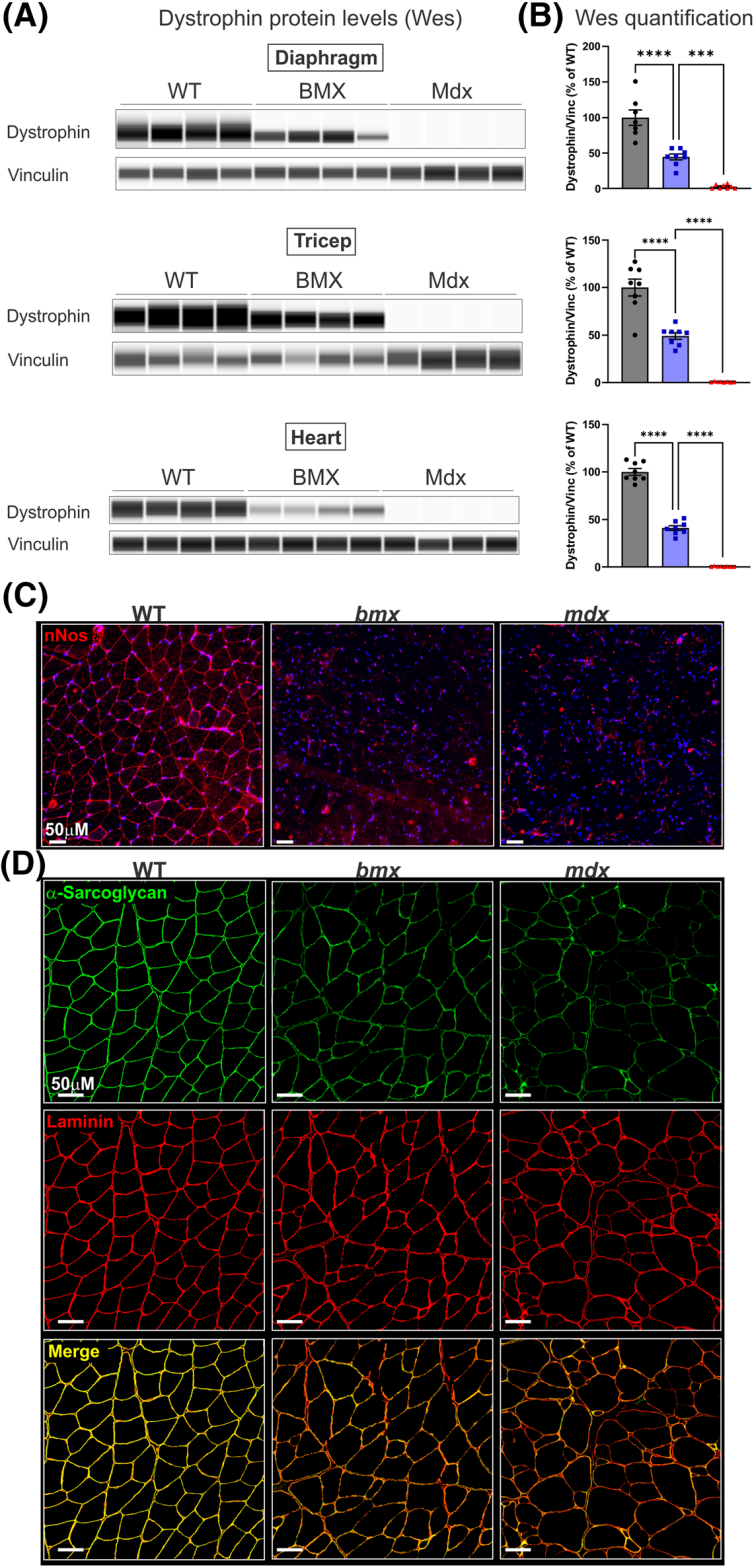
Quantification of dystrophin protein in *bmx* mice using capillary Western assay and localization of dystrophin‐associated proteins. (A) Dystrophin protein levels were determined by capillary electrophoresis immunoassay. Depicted is a virtual Wes blot. (B) Wes quantification in the diaphragm (*P* < 0.0001), triceps (*P* < 0.0001) and heart (*P* < 0.0001) of *bmx* mice. ANOVA, *n* = 7–8. **P ≤ 0.01, ****P ≤ 0.0001. (C) nNOS immunofluorescence (red) was performed in the gastrocnemius muscles and shows an absence of staining for both *bmx* and *mdx*. (D) Immunofluorescence for the dystrophin‐associated protein α‐sarcoglycan (green) was performed in gastrocnemius muscles of WT, *bmx* and *mdx52* muscles. Results show reduced staining and reduced colocalization in *bmx* and *mdx52* at the sarcolemma. Laminin (red) was used to visualize all muscle fibres and DAPI was used as a counterstain to visualize myonuclei. Bar = 50 μM.

We assayed dystrophin protein levels via immunofluorescence and saw visual reductions in *bmx* skeletal and cardiac muscles versus WT (*Figure* 5B). We next performed capillary Western immunoassays (Wes), as dystrophin quantification using this method has proven to be highly sensitive, reproducible and quantitative over a large dynamic range.^28^ Indeed, *bmx* skeletal and heart muscles showed, on average, ~50% less dystrophin than WT (diaphragm, *P* = 0.0002; triceps, *P* < 0.0001, heart, *P* = 0.0001; quadriceps, *P* = 0.0064; TA, *P* = 0.0016; gastroc, *P* = 0.0010) (*Figures* 6A,B and S4). In the diaphragm, we also detected the shorter, C‐terminal, ubiquitously expressed dystrophin isoform Dp71. Consistent with previous reports,^29^ we observed a ~2.5‐fold increase in Dp71 expression in *mdx52* diaphragms (*P* = 0.0445; *Figure* S5). *bmx* showed slightly elevated levels of Dp71 (~2‐fold); however, this did not reach statistical significance (*P* = 0.126; *Figure* S5).

We next queried localization of nNOS and of the dystrophin‐associated protein (DAP) α‐sarcoglycan. Consistent with loss of dystrophin STR R17 (encoded by exon 45), which is necessary for nNOS binding,^30^ *bmx* muscles showed complete loss of nNOS localization at the sarcolemma as did dystrophin‐null *mdx52* muscles (*Figure* 6C). Examining α‐sarcoglycan localization via immunofluorescence we observed both reduced amounts and reduced colocalization at myofibre membranes in both *bmx* and *mdx52* muscle (*Figure* 6D). Collectively, these data show *bmx* exhibit reduced association between dystrophin and DAPs at the sarcolemma; this is likely directly linked with the internal dystrophin truncation (nNOS) and reduced dystrophin levels (α‐sarcoglycan) in *bmx* muscle.

### Bmx muscles express higher levels of inflammatory genes and miRNAs

**Figure 7:**
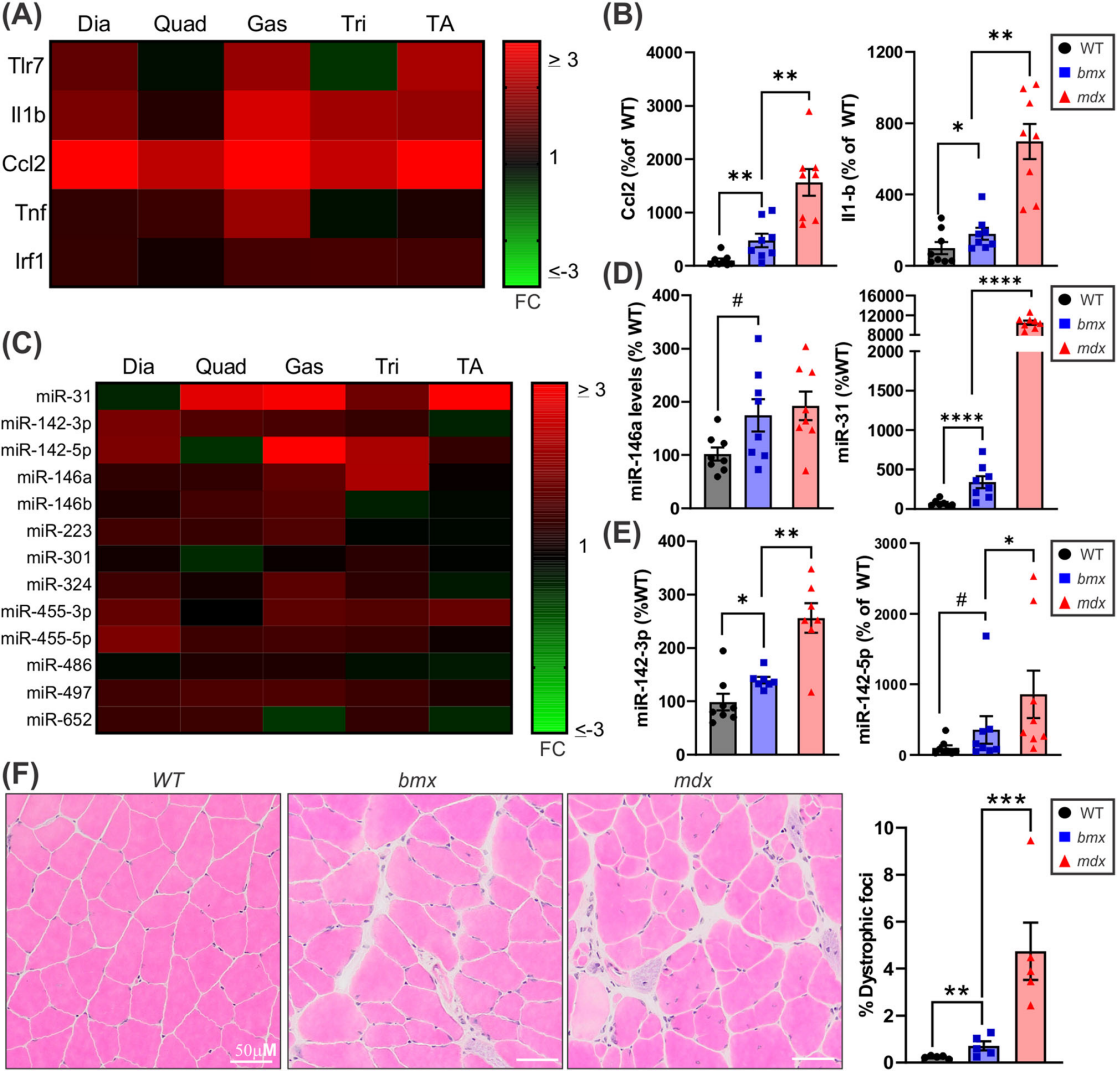
Muscle inflammation is present in *bmx* mice. (A) Heat map of inflammatory gene expression depicting fold change in *bmx* over WT in the diaphragm (Dia), quadriceps (Quad), gastrocnemius (Gas), triceps (Tri) and tibialis anterior (TA). (B) *Ccl2* and *Il1b* are elevated in *bmx* gastrocnemius muscles *n* = 8. (C) Heat map of dystrophin‐targeting miRNA and inflammatory miRNA expression depicting fold change in *bmx* over WT. (D) Graphs show elevated levels of dystrophin‐targeting miRNAs miR‐146a and miR‐31 in *bmx* gastrocnemius muscles *n* = 8. (E) Graphs show elevated levels of chronic inflammatory miRNAs miR‐142‐3p and miR‐142‐5p in *bmx* gastrocnemius muscles. (F) Haematoxylin and eosin‐stained gastrocnemius of WT, *bmx* and *mdx52* mice. Left: representative images, right: quantification of dystrophic foci in muscles showing *bmx* mice have an increase in inflammation and necrosis *n* = 5. ANOVA. #*P* < 0.10; *P < 0.05; **P ≤ 0.01, ****P ≤ 0.0001.

We examined levels of inflammation‐induced genes using a pre‐determined inflammatory panel consisting of *Tlr7*, *Il1b*, *Ccl2*, *Tnf* and *Irf1*. Relative levels of inflammatory transcripts for all *bmx* muscles were determined versus WT and were plotted as a heat map (*Figure* 7A), which demonstrated that the gastroc muscle has the highest levels of inflammatory transcripts and *Ccl2* is the most highly upregulated transcript across all *bmx* muscles. We also plotted individual transcript levels for WT, *bmx* and *mdx52* gastrocs which demonstrated significant increases in *Ccl2* (+375%, *P* = 0.0012) and *Il1b* (+80%, *P* = 0.0140) (*Figure* 7B). We also analysed miRNA panels that we have previously described—dystrophin‐targeting miRNAs (DTMs)^15^ and inflammatory miRNAs,^31^ both regulated by the inflammatory transcription factor NF‐κB. A heat map showing levels of inflammatory‐driven miRNAs in *bmx* versus WT revealed that the gastroc expresses the highest levels of DTMs and inflammatory miRNAs (*Figure* 7C). Plotting individual miRNA expression levels in gastroc demonstrated increased DTMs (miR‐146a, 72% increase, *P* = 0.0928; miR‐31, 265% increase, *P* < 0.0001) and inflammatory miRNAs (miR‐142‐3p, 33.81% increase, *P* = 0.0183; miR‐142‐5p, 255.7% increase, *P* = 0.0583; *Figure* 7D,E).

BMD muscle biopsies show immune cell infiltration and myofibre necrosis.^32^ As *bmx* gastroc muscles were most affected at the molecular level, we next stained sections with haematoxylin and eosin (H&E) and quantified inflammation and necrosis (dystrophic foci). H&E staining visually demonstrated pathology in *bmx*, and quantification showed a 29% increase (*P* = 0.0021) in dystrophic foci as compared with WT (*Figure* 7F).

### Fibrotic gene expression and staining are increased in bmx

**Figure 8:**
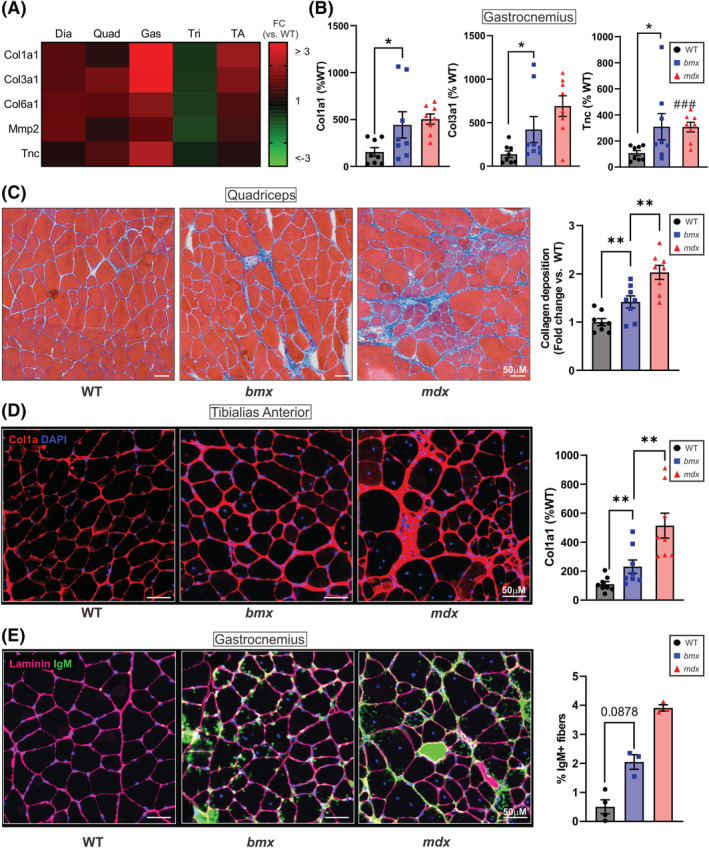
Markers of fibrosis and muscle damage in *bmx* mice. (A) Heat map showing relative levels of fibrosis‐associated genes in all *bmx* skeletal muscle analysed (vs. WT). (B) qRT‐PCR of gastrocnemius muscle from WT, *bmx* and *mdx52* muscles showing elevated Col1a1 (*P* = 0.0288), Col3a1 (*P* = 0.0452) and Tnc (*P* = 0.0273) *n* = 8. (C) Trichrome staining of quadriceps muscle from WT, *bmx* and *mdx52* mice. *Bmx* mice show a 41.7% increase in fibrotic staining area (*P* = 0.0217), ANOVA. (D) left: Col1a1 immunofluorescence was performed in the tibialis anterior (TA) muscles and shows thickening around myofibres in *bmx* and *mdx52* mice; right: qPCR of Col1a1 in TA muscles showed elevated expression in *bmx* (*P* = 0.0085) *n* = 8. (E) WT, *bmx* and *mdx52* quadriceps were immunostained with an antibody against IgM to assess muscle damage. The *bmx* gastrocnemius muscles showed a 309.2% increase in IgM‐positive myofibers (*P* = 0.0878; *n* = 3–4). ANOVA, *P ≤ 0.05, **P ≤ 0.01.

DMD muscles show high levels of fibrosis, and BMD patient muscles show variable fibrosis which inversely correlates with functional measures.^33^ In *mdx52*, fibrosis is apparent in the diaphragm by 8–10 months of age and in other skeletal muscles by 20 months.^34^ Preceding obvious fibrosis, elevated levels of fibrosis‐associated genes and increased deposition of perimysial and endomysial collagen are observed.^35^ We examined a panel of genes indicative of fibrosis signalling including *Col1a1*, *Col3a1*, *Col6a1*, *Mmp2* and *Tnc* ^36^, ^37^ and plotted relative levels in *bmx* (vs. WT) as a heat map for all muscles examined (*Figure* 8A). The *bmx* gastroc showed the highest upregulation of fibrosis‐associated genes (*Figure* 8A,B). Representative graphs of a few fibrosis‐associated transcripts show significant increases in *Col1a1* (+188%, *P* = 0.0288), *Col3a1* (+207%, *P* = 0.0452), and *Tnc* (+188%, *P* = 0.0273) in *bmx* gastroc (*Figure* 8B), as well as *Col3a1* and *Tnc* in the TA (*Figure* S6A). Masson's trichrome staining showed increased collagen deposition in *bmx* versus WT quadriceps (*P* = 0.0083; *Figures* 8C and S6B), and Collagen 1a immunofluorescence showed visually increased collagen around the sarcolemma in *bmx* TA (*Figure* 8D). qPCR supported this observation (*Figure* 8D). Additionally, to assess muscle damage, quadriceps muscle sections were immunostained with an anti‐IgM antibody. *bmx* mice had a visual increase in IgM‐positive myofibres compared with WT mice (*Figures* 8E and S6C; *P* = 0.0878).

## Discussion

Here we describe the characterization of the *bmx* mouse generated using CRISPR/Cas9 to delete exons 45–47 of the endogenous murine *Dmd* gene. To our knowledge, this is the first described murine BMD model. These mice recapitulate several features of BMD and harbour a phenotype intermediate to healthy and *mdx52* mice. Specifically, *bmx* mice show deficits in muscle and cardiac function, reduced dystrophin protein in skeletal and cardiac muscle, and histopathology consistent with moderate dystrophy.

A previous report described generation of the first BMD rat model in which exons 3–16 of the rat *Dmd* gene were deleted using CRISPR/Cas9.^38^ The BMD rat shows muscle inflammation, muscle fibrosis, heart fibrosis and reduced dystrophin protein, but not mRNA. These molecular and histopathological deficits in the Becker rat, however, do not result in muscle function deficits. We have built upon this important first Becker model by generating and characterizing the first murine BMD model, which we refer to as the *bmx* mouse; we find histopathological and molecular deficits similar to the BMD rat and additionally observe reduced muscle function. The *bmx* model described here is thus an important addition to the DMD/BMD field and will enable direct comparisons with *mdx* studies previously performed.

Dystrophinopathy research has focused almost exclusively on DMD because patients completely lack dystrophin protein, which results in a more severe disease. Indeed, four AO exon skipping therapies, which lead to the production of a BMD‐like dystrophin protein, have been approved for DMD. Availability of these drugs, with additional similar drugs in the pipeline, shifts the muscular dystrophy field into a new era where increased numbers of Becker‐like patients will need care. Data from the *bmx* mouse presented here demonstrate that even if dystrophin restoration from exon skipping treatment achieves 100% efficacy, the resultant ‘Becker‐like’ dystrophin may be expressed at lower‐than‐normal levels in muscle due to other factors, and therefore pathology including muscle damage, inflammation, weakness and cardiomyopathy will persist.

Deletion of *DMD* exons 45–47 (coding for 150 amino acids in the rod domain) is the most common BMD‐causing mutation. Previous reports show that this deletion removes STR17, which is necessary for proper nNOS localization.^39^ Because the exon boundaries do not perfectly correlate with the physical boundaries of individual STRs at the protein level, the 45–47 deletion also leads to a disruption or ‘out‐of‐phase’ structure of the rod domain's STR pattern.^40^ Several natural history and longitudinal studies show ‘out‐of‐phase’ mutations lead to more severe disease outcomes. For instance, Bello et al. performed a longitudinal study that categorized BMD patients into groups: deletions beginning at exon 45 ‘del 45’, ending on exon 51 ‘del 51’ or other mutations.^S1^ The ‘del 45’ group showed greater severity in almost every measure as compared with other BMD‐causing mutations and had lower levels of dystrophin protein. A separate study demonstrated that the exon 45–47 deletion and other STR‐disrupting or ‘out‐of‐phase’ deletions result in earlier onset dilated cardiomyopathy.^40^ The phenotype we have described in the *bmx* mouse here is consistent with a ‘more severe’ Becker‐like phenotype.

To understand the potential implications of DMD exon skipping and Becker‐like dystrophin isoforms, much work has focused on natural history data from BMD patients that model the ‘result’ of skipping DMD exons 45, 51 and 53,^41^ ^,^^S2^ (Clinical Trial #NCT01539772). Data presented here from *bmx* mice (model for exon 45 skipping) further contributes to this body of knowledge*. bmx* mice exhibit significant weakness and cardiac dysfunction despite substantial improvement in pathology over dystrophin‐null mice. This supports the idea that exon skipping will slow DMD disease progression, but not completely ameliorate symptoms and pathology. Moving forward, creating additional Becker mouse models for comparison with the *bmx* mouse will help us to understand which truncated dystrophin isoforms will result in the best outcomes, both for BMD patients and for DMD patients with mutations amenable to exon skipping.

Despite a substantial improvement in pathology over dystrophin‐null mice, we show here that *bmx* mice expressing 100% of a truncated in‐frame dystrophin transcript still develop muscle weakness and cardiac dysfunction. In previous murine exon skipping studies, some reports show dystrophin protein levels as low as 30% can improve skeletal muscle degeneration.^S3^ Other reports show that ~20% of dystrophin protein expression improves symptoms and that as little as 4% is sufficient to provide some benefit to muscle function and survival.^S4–S7^ This brings up the much‐debated question: ‘How much dystrophin protein is enough?’ Data presented here suggests that while 20–30% dystrophin protein will significantly improve disease severity, even if 100% of *DMD* dystrophin transcripts are skipped and therefore in‐frame, some muscle damage, inflammation and weakness are still likely because dystrophin levels will be substantially lower than what is seen in healthy muscle.

Interestingly we see reduced dystrophin protein, but not mRNA, in *bmx* skeletal and cardiac muscle. This is consistent with our previous report showing reduced and variable dystrophin protein, but not mRNA, in BMD patient biopsies that harbour a deletion in *DMD* exons 45–47.^15^ In prior reports, we described dystrophin‐targeting miRNAs (DTMs) that reduce dystrophin protein by binding to the dystrophin 3′ UTR.^15^, ^41^ Interestingly, in *bmx* muscles, we observe elevated levels of DTMs, with miR‐146a and miR‐31 being the most consistently up‐regulated. It is likely that these miRNAs function to reduce dystrophin protein levels in *bmx* muscles. To definitively demonstrate this, however, future experiments in *bmx* mice will require targeting/inhibiting individual miRNAs, or administering DTM‐reducing drugs, followed by a detailed assessment of changes in dystrophin protein levels. *bmx* muscles also have elevated levels of miRNAs from an ‘inflammatory miRNA’ panel that we previously reported.^31^ These miRNAs can ‘turn on’ NF‐κB signalling, activating a positive feedforward loop of inflammation. The collective increase in these pathological miRNAs (DTMs and inflammatory miRNAs) coupled with the reduction in dystrophin protein likely exacerbates inflammation in *bmx* muscles. We have demonstrated a similar mechanism in myositis where we observe reduced dystrophin and increased pathological miRNAs.^41^ In early reports, others also noted abnormal and lower levels of dystrophin in human myositis biopsies.^S8^ Further work to determine if this miRNA–dystrophin–inflammation axis is driving disease in other inflammatory muscle disorders will be important, especially given that elevated levels of these miRNAs are found in other muscle disorders.

The observation of slightly elevated Dp71 expression in the diaphragm is interesting, as a previous study showed increased Dp71 can have a dominant negative effect by displacing full‐length dystrophin and further increasing muscle damage.^29^ ^,^^S9^ Further, a recent report showed Dp71 overexpression can have detrimental effects on cardiac function.^S10^ Though we did not observe a strong signal via Wes in the heart, it should be noted that high molecular weight capillaries that were used to resolve full‐length dystrophin may have prevented full resolution of Dp71 signal in cardiac or other tissues. In future studies, it would be interesting to look at cardiac‐specific expression over the lifetime of *bmx*.

Cardiac involvement is present in 70–80% of BMD patients and is a leading cause of mortality for this population.^40^ Specifically, it has been reported that patients can present with arrhythmias, a decline in ejection fraction and dilated cardiomyopathy (DCM) similar to DMD patients.^S11^ Interestingly, one study reported a significantly earlier DCM onset in BMD patients with an exon 45–47 deletion (median age of 27 years) versus patients with a 45–48 or 45–49 deletion (median ages of 38 and 41 years, respectively).^S12^ Here we utilized echocardiography to measure heart function in aged *bmx* mice. We find *bmx* mice develop cardiac dysfunction characterized by a decrease in left ventricular ejection fraction and fractional shortening, consistent with BMD patients. In the clinic, BMD patients are also found to develop regional wall motion abnormalities, late gadolinium enhancement and reduced global strain. In future studies, it will be important to further characterize the life history of *bmx* hearts, identify preclinical changes, discover useful biomarkers for preclinical drug testing and determine the impact of various stressors on *bmx* cardiomyopathy.

Glucocorticoid treatment is a proven and FDA‐approved intervention for DMD, which is associated with delayed loss of ambulation.^S13^ However, glucocorticoid treatment is not commonly used for BMD and has an unclear risk–benefit ratio due to the safety concerns of chronic glucocorticoid treatment over the longer BMD disease course; thus far, the BMD population has avoided widespread glucocorticoid use due to a non‐tolerability of glucocorticoid side effects such as weight gain, diabetes, muscle atrophy, bone fragility, adrenal suppression and mood disturbances. However, because we have observed increased NF‐κB‐driven inflammation, as measured by both expression analysis of genes such as Ccl2 and by quantification of H&E staining, it suggests that BMD patients could benefit from anti‐inflammatory drug treatment regimens. Moving forward, the *bmx* mouse will enable us to look at how efficacy and safety profiles impact Becker‐like phenotypes using both traditional glucocorticoids (prednisone and deflazacort) and next‐generation drugs with improved safety profiles, such as vamorolone.^36^, ^42^ This can inform both current clinical management and future drug development for Becker patients.

There are several promising treatments in clinical trials for BMD. These include EDG‐5506 (Edgewise Therapeutics) and vamorolone (ReveraGen BioPharma). Excitingly, EDG‐5506 showed improvements in muscle damage biomarkers in a Phase I study of treated BMD patients.^S14^ We hope that the generation of the *bmx* mouse will further advance treatments for BMD patients and enable the elucidation of the mechanism of action of treatments.

In conclusion, our characterization of novel *bmx* mice here demonstrates the utility of such a model to the DMD/BMD field. Moving forward, this model will be useful for conducting preclinical studies to test novel therapeutic targets or repurposed drugs for BMD and for identifying biomarkers of disease progression. As a follow‐up to these studies, there is a need to generate more murine BMD models to better understand BMD disease progression, to test functionality of Becker‐like dystrophin isoforms and to test various interventions that would benefit BMD patients and exon skipping‐treated DMD patients.

## Conflicts of interest

AJR is co‐founder and chief scientific officer of Edgewise Therapeutics. BNS is an employee of Edgewise Therapeutics. Edgewise Therapeutics did not fund the research and did not fund the generation or initial characterization of the *bmx* mouse model; AJR and BNS performed and analysed *ex vivo* muscle contraction experiments. CRH and AAF have filed a provisional intellectual property application related to the research in the manuscript.

## Supporting information


**Figure S1.** **Validation of *bmx* mice.** Using a probe against dystrophin exons 45–46 validates deletion of this region in the tibialis anterior (*p* < 0.0001), diaphragm (*p* < 0.0001), and heart (*p* < 0.0001). *n* = 7–8. ANOVA ****p ≤ 0.0001
**Figure S2. *bmx* have reduced muscle force**. (a) *In vivo* maximum specific isometric torque (left) and specific isometric torque‐frequency curve (right) for anterior crural muscles of WT, *bmx*, and *mdx52* mice. (b) Ex vivo EDL isometric force drop after 10 lengthening contractions with eccentric force curve for each of 10 lengthening contractions. ANOVA; *p < 0.05.
**Figure S3. Increased mass of skeletal muscle in *bmx* mice.** Mass of the gastrocnemius (*p* = 0.0143) and triceps (*p* = 0.0583) is increased in *bmx* mice. *n* = 12. ANOVA, *p ≤ 0.05, ****p ≤ 0.0001
**Figure S4. Reduced dystrophin protein in *bmx* skeletal muscle*.*** Dystrophin protein levels were determined by capillary electrophoresis (Wes). (a‐c) Dystrophin protein levels were reduced in the quadriceps (*p* = 0.0016), tibialis anterior (*p* = 0.0003), and gastrocnemius (*p* < 0.0001) in *bmx* mice. *n* = 7–8. ANOVA, **p ≤ 0.01, ***p < 0.001, ****p ≤ 0.0001
**Figure S5. Dystrophin isoform Dp71 is slightly increased in *bmx* and significantly increased in *mdx*.** Dystrophin Dp71 protein levels were determined by capillary electrophoresis (Wes). (a) *Left*; Virtual blot of Dp71 levels in the diaphragm, *Right*; quantification of Wes signal (WT vs. mdx *p* < 0.0489; WT vs. *bmx p* = 0.126. *n* = 3–4). ANOVA, *p ≤ 0.05, ***p < 0.001, ****p ≤ 0.0001. One outlier capillary did not exhibit a signal and was removed from the WT cohort. (B) Wes electropherogram of WT, *bmx* and *mdx* signal.
**Figure S6. Markers of fibrosis and muscle damage in *bmx* mice.** (a) qRT‐PCR of tibialis anterior muscle from WT, *bmx* and *mdx* muscles showing elevated Col3a1 (*p* = 0.0167), Col6a1 (*p* = 0.0205), and Tnc (*p* = 0.0178). (b) Trichrome staining of quadriceps muscle from WT, *bmx*, and *mdx* mice. (c) WT, *bmx*, and *mdx* TAs were stained with an antibody against IgM to assess muscle damage. The *bmx* TA muscles show a trend of (*p* = 0.0755) increase in IgM‐positive myofibers (*p* = 0.0878; *n* = 4). ANOVA, *n* = 8 for a; t‐test for c. *p ≤ 0.05, ***p < 0.001, ****p ≤ 0.0001
