# Supplementary material for: The X‐linked Becker muscular dystrophy (bmx) mouse models Becker muscular dystrophy via deletion of murine dystrophin exons 45–47
Source: J Cachexia Sarcopenia Muscle. 2023 Jan 11;14(2):940–54. doi: 10.1002/jcsm.13171 (PMC10067474; doi:10.1002/jcsm.13171)
Supplement: Supplementary file 1 — Figure S1. Validation of bmx mice. Using a probe against dystrophin exons 45–46 validates deletion of this region in the tibialis anterior (p < 0.0001), diaphragm (p < 0.0001), and heart (p < 0.0001). n = 7–8. ANOVA ****p ≤ 0.0001 Figure S2. bmx have reduced muscle force. (a) In vivo maximum specific isometric torque (left) and specific isometric torque‐frequency curve (right) for anterior crural muscles of WT, bmx, and mdx52 mice. (b) Ex vivo EDL isometric force drop after 10 lengthening contractions with eccentric force curve for each of 10 lengthening contractions. ANOVA; *p < 0.05. Figure S3. Increased mass of skeletal muscle in bmx mice. Mass of the gastrocnemius (p = 0.0143) and triceps (p = 0.0583) is increased in bmx mice. n = 12. ANOVA, *p ≤ 0.05, ****p ≤ 0.0001 Figure S4. Reduced dystrophin protein in bmx skeletal muscle. Dystrophin protein levels were determined by capillary electrophoresis (Wes). (a‐c) Dystrophin protein levels were reduced in the quadriceps (p = 0.0016), tibialis anterior (p = 0.0003), and gastrocnemius (p < 0.0001) in bmx mice. n = 7–8. ANOVA, **p ≤ 0.01, ***p < 0.001, ****p ≤ 0.0001 Figure S5. Dystrophin isoform Dp71 is slightly increased in bmx and significantly increased in mdx. Dystrophin Dp71 protein levels were determined by capillary electrophoresis (Wes). (a) Left; Virtual blot of Dp71 levels in the diaphragm, Right; quantification of Wes signal (WT vs. mdx p < 0.0489; WT vs. bmx p = 0.126. n = 3–4). ANOVA, *p ≤ 0.05, ***p < 0.001, ****p ≤ 0.0001. One outlier capillary did not exhibit a signal and was removed from the WT cohort. (B) Wes electropherogram of WT, bmx and mdx signal. Figure S6. Markers of fibrosis and muscle damage in bmx mice. (a) qRT‐PCR of tibialis anterior muscle from WT, bmx and mdx muscles showing elevated Col3a1 (p = 0.0167), Col6a1 (p = 0.0205), and Tnc (p = 0.0178). (b) Trichrome staining of quadriceps muscle from WT, bmx, and mdx mice. (c) WT, bmx, and mdx TAs were stained with an antibody against IgM to [file JCSM-14-940-s001.docx]

**Supporting Information**

The X-linked Becker muscular dystrophy (*bmx*) mouse models Becker muscular dystrophy via deletion of murine dystrophin exons 45-47

Christopher R. Heier^1,2^, Nikki M. McCormack^1^, Christopher B. Tully^1^, James S. Novak^1,2^, Breanne L. Newell-Stamper^3^, Alan J. Russell^3^, Alyson A. Fiorillo^1,2^

^1^Center for Genetic Medicine Research, Children’s National Hospital, Washington, District of Columbia, USA

^2^Department of Genomics and Precision Medicine, George Washington University School of Medicine and Health Sciences, Washington, District of Columbia, USA

^3^Edgewise Therapeutics, BioFrontiers Institute, University of Colorado, Boulder, CO 80303, USA.

*Corresponding author:

Alyson A. Fiorillo, PhD

Email: afiorillo@childrensnational.org

Tel: 202-545-2813

**Supporting Methods**

*Motor function tests*

Wire Hang: For two-limb wire hang, a wire hanger was suspended ~35cm above a cage with soft bedding. Mice were hung using only their forelimbs; however, they were allowed to swing and hang with all four limbs if able. Hang time was recorded, with 600 seconds used as a cutoff.

*Box hang:* For four-limb grid hang tests the same parameters were used as in wire hang, but mice instead hung upside down from a handmade box covered in wire mesh (1x1cm grid). *n* = 8 per group were used for analysis.

*In vivo Isometric Torque*

To measure *in vivo* torque production of the anterior crural muscles (TA, extensor digitorum longus (EDL), peroneus tertius, and extensor hallucis longus), mice were anesthetized with 1.5% isoflurane-mixed O_2_ and hair removed from lower hind limbs, while the foot was attached to the dual-mode lever and maintained at 90° for isometric torque assessment (Aurora Scientific) as detailed in [1]. Isometric muscle contractions were stimulated at 1.0–2.0 mA using Pt-Ir needle electrodes inserted percutaneously adjacent to the peroneal nerve. Peak isometric torque was measured in response to tetanic stimulations at 20, 40, 60, 80, 100, 120, 140, 160, 180, and 200 Hz, providing a 60s rest period between stimuli.  *n* = 5-7 per group were used for analysis.

*Ex vivo eccentric contractions*

An eccentric injury protocol was performed in male *bmx, mdx52* and WT mice based on previously reported protocols [2]. Mice were anesthetized via inhaled isoflurane gas mixture (~2.1%) and placed ventrally for surgery. A superficial incision was made just below the ankle joint to just above the knee joint and the skin removed. The leg was pinned through the foot and thigh with the anterior portion of the leg exposed for removal of the intact EDL. Throughout the procedure, skinned portions of the limb were regularly bathed in Krebs mammalian ringer solution bubbled with a gas mixture of 5% CO_2_ and 95% O_2_. Loops of 5-0 suture were tied around proximal and distal EDL tendons and were mounted in a water-jacketed bath of Ringers solution maintained at 27⁰C with continuously infused CO_2_/O_2_ gas mixture. The distal end of the muscle was connected to a rigid mounting point, while proximal ends were hooked onto a combination servomotor and force transducer (Aurora 300C-LR).

A force-frequency sweep of isometric stimulations at frequencies ranging from 25-175 Hz were applied in 25 Hz increments one minute apart to characterize baseline contractile properties of the EDL. The force-frequency was followed by three 100 Hz stimulations spaced at 10-minute increments to verify preparation stability. After the third 100 Hz stimulation, the protocol moved into the injury phase. A single 100 Hz isometric stimulation was performed to verify preparation stability again, and after a 1 min rest, 10 eccentric contractions were performed 1 minute apart. Each eccentric contraction consisted of 100 ms isometric stimulation at 100 Hz, followed by active lengthening with a magnitude of 0.1 L0 at a rate of 2 L0/s, where L0 is the resting length of muscle at which it produces maximal isometric force. Total active lengthening time was 50 ms. After lengthening, muscles were held at 1.1 L0 with stimulation for an additional 100 ms. Data acquisition and processing were performed using custom software written by Benjamin D. Robertson. Force loss of the EDL muscle was calculated across the 10 eccentric contractions as a percent of pre-injury force. An ANOVA with a Holms-Sidak post-hoc analysis (WT vs. *bmx*, *bmx* vs. *mdx52*) was performed on the calculated force loss (WT *n* = 6; *bmx n* = 6, *mdx52 n* = 6).

*Echocardiography*

Echocardiography was performed as previously described [3]. Briefly, aged (18-month-old) WT, *bmx* and *mdx52* mice (*n* = 5-7) were assayed using a Vevo 3100 micro-ultrasound imaging system (VisualSonics). Images were acquired via high resolution electrocardiogram-gated kilohertz visualization (EKV) and via M-mode imaging of the parasternal long axis and the parasternal short axis. Image analysis and calculation of cardiac ejection fraction and fractional shortening was performed using Vevo software.

*Serum creatine kinase*

Blood was collected via retro-orbital route from aged (one-year old) WT, *bmx* and *mdx52* mice anesthetized under isoflurane then allowed to clot at room temperature for 45 minutes. After centrifugation, serum was collected and stored at -80°C until assay.

*Immunofluorescence*

Anti-dystrophin 1:150 (Abcam, #ab154168), anti-laminin-2 1:100 (Sigma-Aldrich, Cat. #L0663), Anti-Alpha-sarcoglycan 1:100 (Leica Biosystems Cat. #NCL-L-a-SARC), Anti-nitric oxide synthase 1:1000 (Millipore Sigma Cat #N7280) anti-mouse IgM-FITC 1:100 (Sigma Aldrich, #F9259), or anti-collagen 1:100 (Abcam, #ab21286). Secondary antibodies included: goat anti-rabbit 568 1:400 (ThermoFisher, #A-11036), donkey anti-rat 488 1:400 (ThermoFisher, #A-21208), and goat anti-rat 647 1:400 (ThermoFisher, #A-21247).

*BrdU staining*

BrdU staining was performed as previously described [4]. Briefly, frozen slides were thawed for 1 hour at RT and fixed in ice-cold acetone for 10 minutes at -20ºC. Slides were washed twice with PBS for 5 minutes with gentle shaking then incubated in 2N HCl for 30 minutes at 37ºC in a humified chamber to denature DNA. Slides were washed once with PBST for 5 minutes with gently shaking and HCl was neutralized with 0.15 M sodium borate for 10 minutes. Slides were washed three times with PBST for 5 minutes then blocked for 1 hour at RT in a humified chamber. Primary antibodies were diluted in a solution containing 50% 1X PBST and 50% of the blocking solution (anti-BrdU 1:100, anti-laminin 1:100). Slides were incubated in primary antibodies overnight at 4ºC in a humid chamber. The next day, slides were washed 3 times with PBST for 10 minutes each with gentle agitation. Secondary antibodies were diluted in a solution containing 50% 1X PBST and 50% of the blocking solution (Streptavidin 594 1:500, goat anti-rat 488 1:400). Slides were incubated in secondary antibodies for 1.5 hours at room temperature in the dark and then washed 3 times for 10 minutes each with gentile agitation. Slides were incubated with 1.5 μM DAPI in PBS for 10 minutes at RT then washed 1x with PBS. Coverslips were mounted to slides using Prolong Gold Mounting Medium. Slides were immediately imaged using an Olympus VS-120 scanning microscope at 20X.

*Masson’s trichrome staining*

Briefly, frozen muscle sections were thawed for 30 minutes at room temperature, fixed in 4% paraformaldehyde for 1 hr at room temperature, and re-fixed in Bouin’s solution overnight at room temperature. Slides were washed for 1-2 minutes in running tap water and briefly rinsed in deionized water. Nuclei were stained by incubating sections in equal parts of Hematoxylin Solution A and Solution B for 5 minutes at room temperature followed by rinsing under warm running tap water for 10 minutes and 1 minute in deionized water. Cytoplasm was stained by incubating sections in Biebrich Scarlet-Acid Fuchsin Solution for 5 minutes. Slides were washed 3 times for 1 minute with deionized water and then incubated in phosphotungstic / phosphomolybdic acid solution for 10 minutes. Collagen was stained by incubating slides in Aniline Blue Solution for 5 minutes followed by 3 washes with deionized water for 1 minute. Sections were incubated with 1% Glacial Acetic Acid for 2 minutes followed by 2 washes with deionized water for 1 minute. Sections were dehydrated successively in 70%, 90%, and 100% ethanol for 3 minutes each and then in xylene for 5 minutes. Coverslips were mounted with permount. Slides were imaged using an Olympus VS-120 scanning microscope at 20X.

To analyze images for fibrosis, the Colour Deconvolution 2 plugin for ImageJ was used to separate the dyes [5]. The image containing Aniline blue staining was thresholded, the muscle section was outlined, and the percent of the area of Aniline blue staining (fibrotic area) was determined using the measure tool in ImageJ.

*qPCR*

qPCR was performed as previously reported [6], ~50-100 sections (8mM thickness) of mouse muscle were homogenized in 1mL Trizol (Life Technologies) using a TissueRupter II homogenizer (Qiagen).

*miRNAs.* Total RNA was converted to cDNA using multiplexed RT primers and High-Capacity cDNA Reverse Transcription Kit (ThermoFisher; Carlsbad, CA). miRNAs were then quantified using individual TaqMan assays on an ABI QuantStudio 7 real time PCR machine (Applied Biosystems). Assay IDs include: miR-146a 000468, miR-146b 001097, miR-223 002295, miR-31 00185, miR-320a 002277, miR-382 000572, miR-374a 000563, miR-142-3p 000464, miR-142-5p 002248, miR-301a 000528, miR-324-3p 002509, miR-455-3p 002455, miR-455-5p 001280, miR-497 001346, miR-652 002352, sno202 001232, RNU48 001006.

*mRNAs.* RNA was isolated from muscle using TRIzol. cDNA was synthesized from 1000 ng RNA using a High-Capacity Reverse Transcription Kit (Thermo Fisher Cat. #4368813). qRT-PCR analysis was performed using TaqMan Fast Advanced Master Mix (Thermo Fisher Cat. #4444557) and TaqMan probes (all Thermo Fisher). miRNAs were then quantified using individual TaqMan assays on an ABI QuantStudio 7 real time PCR machine (Applied Biosystems). Assay IDs include: Ccl2 Mm00441242_m1; Il1b Mm00434228_m1 Il1b, Tlr7 Mm00446590_m1, Tnf Mm00443258_m1, Irf1 Mm01288580_m1, Col1a1 Mm00801666_g, Col3a1 Mm01254476_m1, Col6a1 Mm00487160_m1, Tnc Mm00495662_m1, Mmp2 00439498_m1, Hrpt Mm01545399_m1, 18S rRNA Mm03928990_g1.

*Capillary western immunoassay (Wes)*

In each capillary 0.2mg/mL protein was loaded for analysis with antibodies to the dystrophin C-terminus (Abcam #ab15277; 1:15) or vinculin (Abcam #ab130007; 1:100), and anti-rabbit secondary (ProteinSimple #042-206). Compass for SW software was used to quantify chemiluminescence. *n* = 8 per group were used for analysis.

*Muscle Histology Analysis*

Cross-sectional area (CSA) and minimum Feret’s diameter were determined using the MuscleJ macro for Fiji [7] (n=4-6). Variance coefficients for CSA and minimum Feret’s diameter were calculated by dividing standard deviation by the average and multiplying by 1000 (Treat NMD SOP DMD_M.1.2.001). Centrally nucleated fibers were counted manually. Total muscle section area was determined using ImageJ. Total number of myofibers was normalized to total muscle section area. Percent IgM-positive myofibers was calculated to quantify muscle damage. *n* = 3-6 were used for analysis.

Masson Trichrome staining was performed to assay fibrosis (Abcam, #ab150686) according to the Treat-NMD SOP (MDC1A_M.1.2.003). Hematoxylin and Eosin staining was performed to assay tissue morphology and pathology. To analyze necrosis and inflammation, the Colour Deconvolution 2 plugin for ImageJ was used to separate Hematoxylin and Eosin dyes [5]. Eosin staining was thresholded, the muscle section outlined, and the percentage area without eosin stain determined.

*Statistical analysis*

Statistical analyses were performed using GraphPad Prism v.9.0.0 (GraphPad Software, Inc.). One-way ANOVA was performed with Holm-Sidak post-hoc test specifically comparing WT vs. *bmx*, and *bmx* vs. *mdx52* groups. For all graphs, data are presented as mean ± SEM.

**Supporting Figures**

**
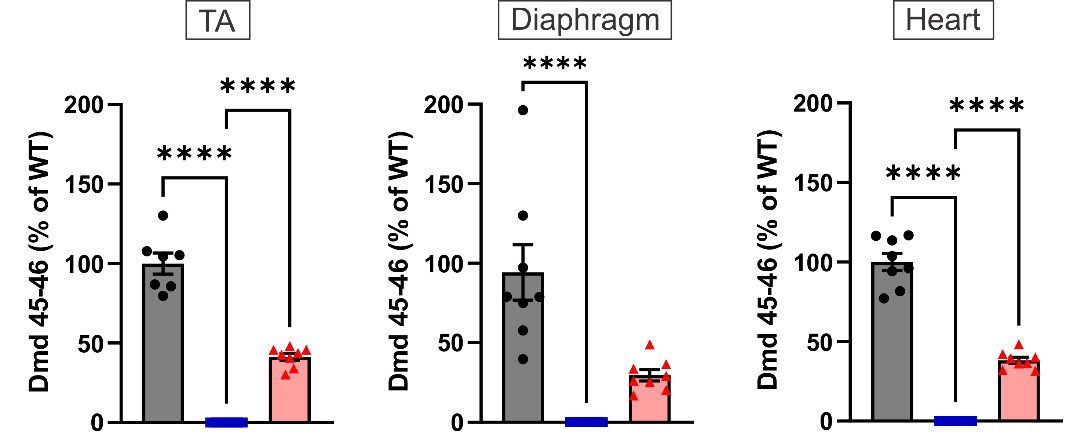
**

**Supporting Information Fig. 1 Validation of *bmx* mice.** Using a probe against dystrophin exons 45-46 validates deletion of this region in the tibialis anterior (TA; *p* < 0.0001), diaphragm (*p* < 0.0001), and heart (*p* < 0.0001). *n* = 7-8. ANOVA *****P* ≤ 0.0001


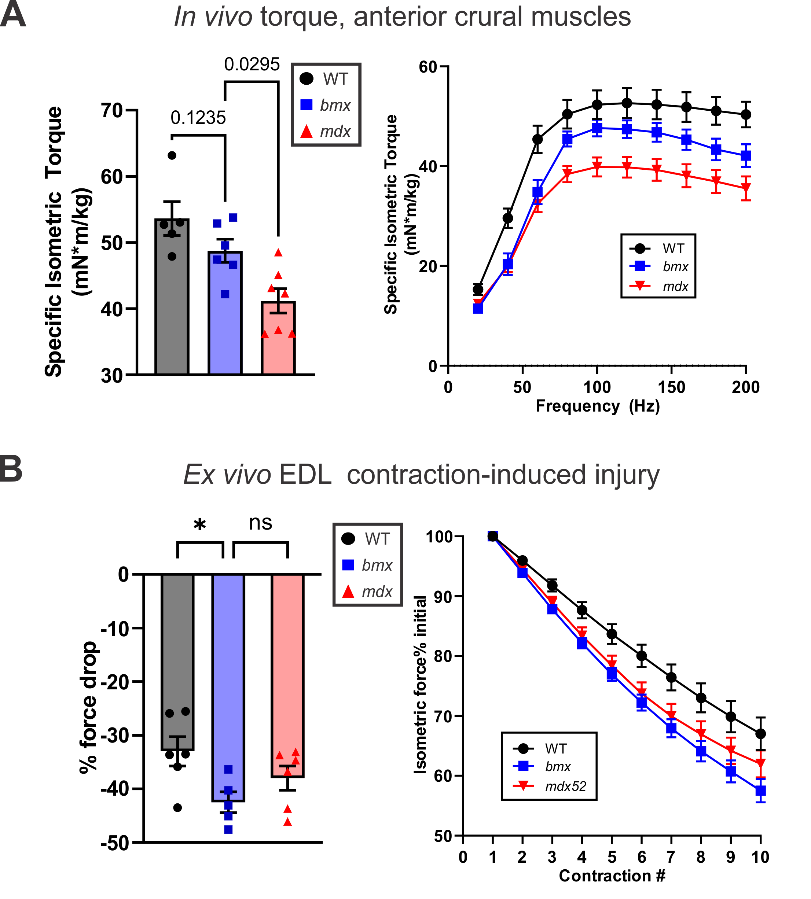


**Supporting Information Fig 2. *bmx* have reduced muscle force**. (a) *In vivo* maximum specific isometric torque (left) and specific isometric torque-frequency curve (right) for anterior crural muscles of WT, *bmx*, and *mdx52* mice. (b) *Ex vivo* EDL isometric force drop after 10 lengthening contractions with eccentric force curve for each of 10 lengthening contractions. ANOVA, **P* < 0.05.


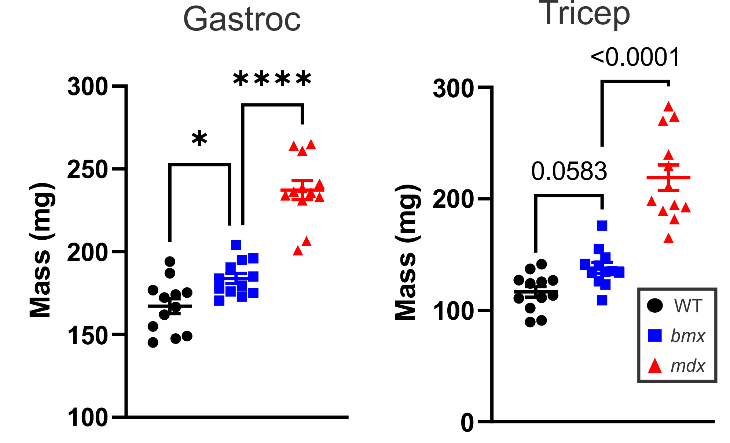


**Supporting Information Fig. 3. Increased mass of skeletal muscle in *bmx* mice.** Mass of the gastrocnemius (*P* = 0.0143) and triceps (*P* = 0.0583) is increased in *bmx* mice. *n* = 12. ANOVA, **P* ≤ 0.05, *****P* ≤ 0.0001


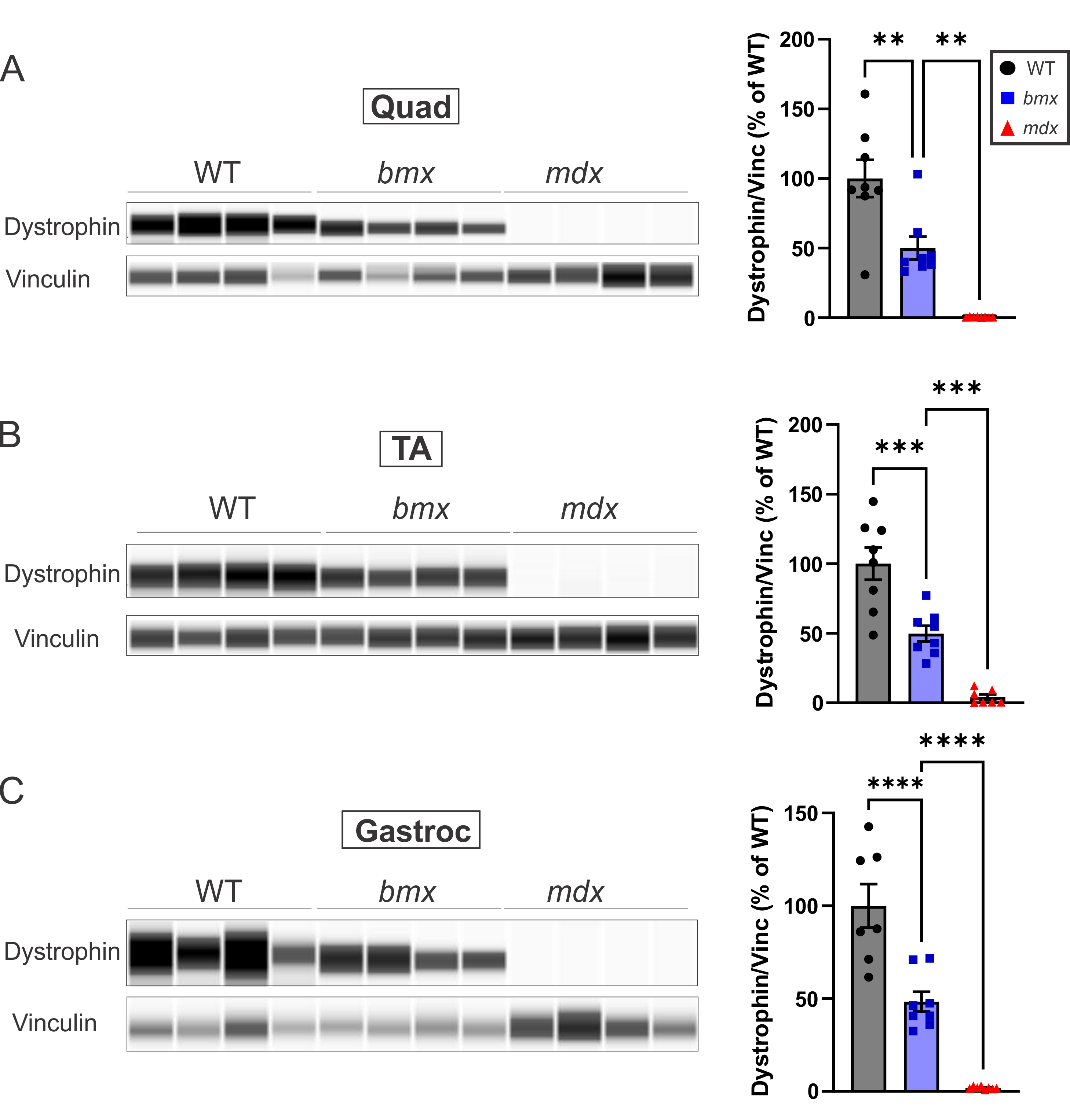


**Supporting Information Fig. 4. Reduced dystrophin protein in *bmx* skeletal muscle*.*** Dystrophin protein levels were determined by capillary western immunoassay (Wes). (a-c) Dystrophin protein levels were reduced in the quadriceps (*P* = 0.0016), tibialis anterior (*P* = 0.0003), and gastrocnemius (*P* <0.0001) in *bmx* mice. *n* = 7-8. ANOVA, ***P* ≤ 0.01, ****P* < 0.001, *****P* ≤ 0.0001

**
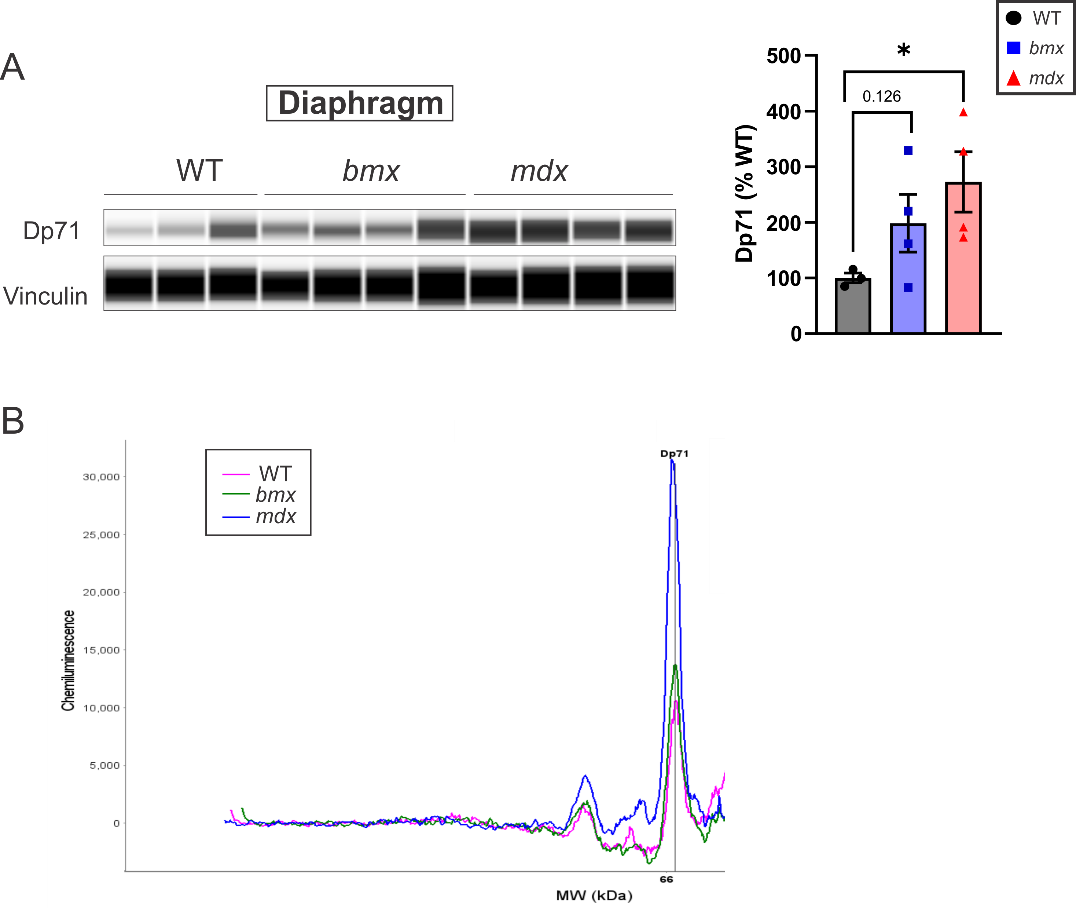
**

**Supporting Information Fig. 5. Dystrophin isoform Dp71 is slightly increased in *bmx* and significantly increased in *mdx*.** Dystrophin Dp71 protein levels were determined by capillary western immunoassay (Wes). (A) *Left*; Virtual blot of Dp71 levels in the diaphragm, *Right*; quantification of Wes signal (WT vs. *mdx* *P* < 0.0489; WT vs. *bmx* *P* = 0.126. *n* = 3-4). ANOVA, **P* ≤ 0.05. One outlier capillary did not exhibit a signal and was removed from the WT cohort. (B) Wes electropherogram of WT, *bmx* and *mdx* signal.

**
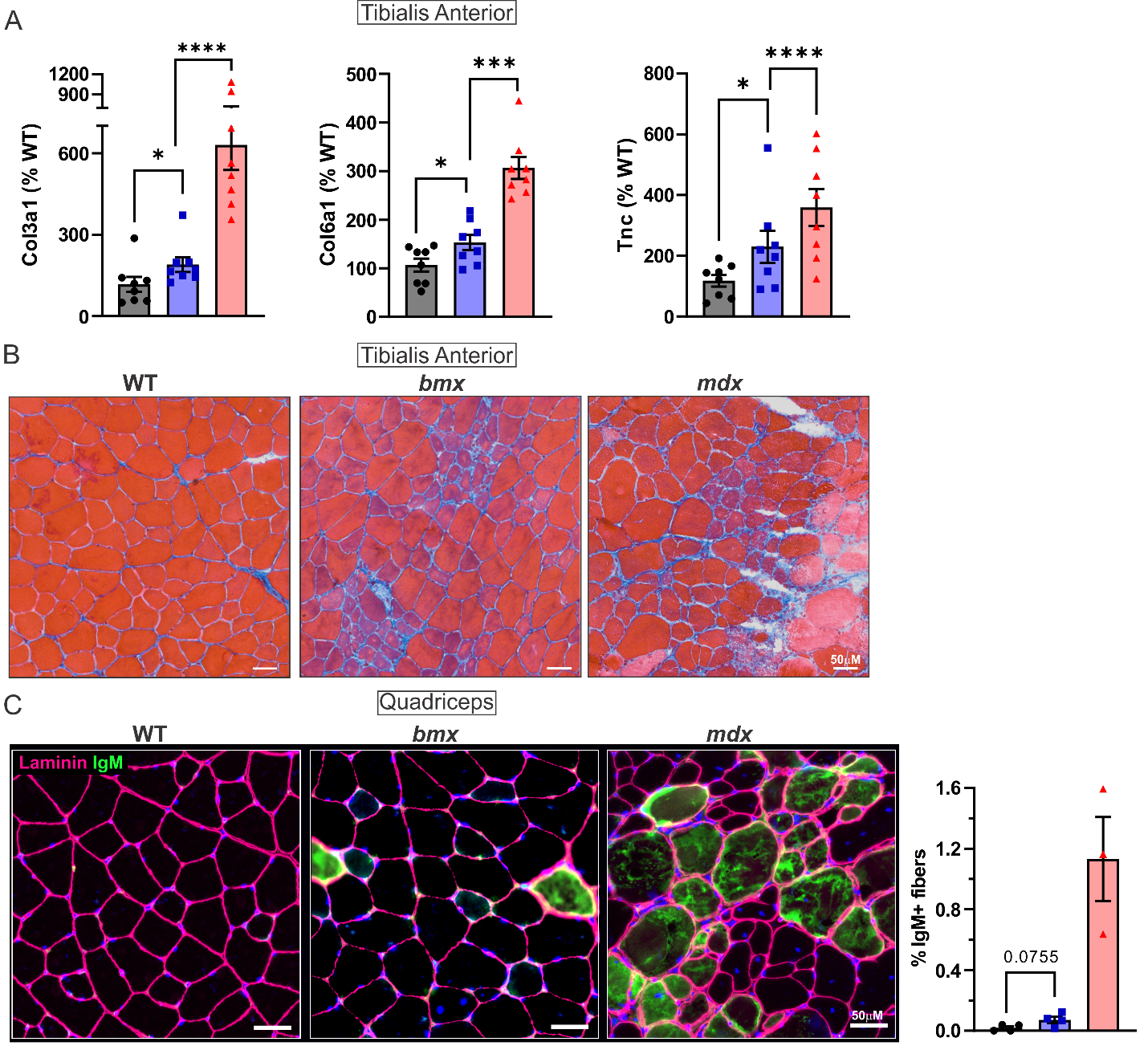
**

**Supporting Information Fig. 6. Markers of fibrosis and muscle damage in *bmx* mice.** (A) qRT-PCR of tibialis anterior muscle from WT, *bmx* and *mdx* muscles showing elevated *Col3a1* (*P* = 0.0167), *Col6a1* (*P* = 0.0205), and *Tnc* (*P* = 0.0178). (B) Trichrome staining of quadriceps muscle from WT, *bmx*, and *mdx* mice. (C) WT, *bmx*, and *mdx* TAs were stained with an antibody against IgM to assess muscle damage. The *bmx* TA muscles show a trend of (*P* = 0.0755) increase in IgM-positive myofibers (*P* = 0.0878; *n* = 4). ANOVA, *n* = 8 for (A); t-test for (C). **P* ≤ 0.05, ****P* < 0.001, *****P* ≤ 0.0001.

**Supporting References**

S1. Bello L, Campadello P, Barp A, Fanin M, Semplicini C, Soraru G, et al. Functional changes in Becker muscular dystrophy: implications for clinical trials in dystrophinopathies. Sci Rep. 2016;6:32439.

S2. Clemens PR, Niizawa G, Feng J, Florence J, D'Alessandro AS, Morgenroth LP, et al. The CINRG Becker Natural History Study: Baseline characteristics. Muscle Nerve. 2020;62:369-76.

S3. Neri M, Torelli S, Brown S, Ugo I, Sabatelli P, Merlini L, et al. Dystrophin levels as low as 30% are sufficient to avoid muscular dystrophy in the human. Neuromuscul Disord. 2007;17:913-8.

S4. Phelps SF, Hauser MA, Cole NM, Rafael JA, Hinkle RT, Faulkner JA, et al. Expression of full-length and truncated dystrophin mini-genes in transgenic mdx mice. Human molecular genetics. 1995;4:1251-8.

S5. Li D, Yue Y, Duan D. Preservation of muscle force in Mdx3cv mice correlates with low-level expression of a near full-length dystrophin protein. Am J Pathol. 2008;172:1332-41.

S6. Li D, Yue Y, Duan D. Marginal level dystrophin expression improves clinical outcome in a strain of dystrophin/utrophin double knockout mice. PLoS One. 2010;5:e15286.

S7. van Putten M, Hulsker M, Nadarajah VD, van Heiningen SH, van Huizen E, van Iterson M, et al. The effects of low levels of dystrophin on mouse muscle function and pathology. PLoS One. 2012;7:e31937.

S8. Sewry CA, Clerk A, Heckmatt JZ, Vyse T, Dubowitz V, Strong PN. Dystrophin abnormalities in polymyositis and dermatomyositis. Neuromuscul Disord. 1991;1:333-9.

S9. Leibovitz S, Meshorer A, Fridman Y, Wieneke S, Jockusch H, Yaffe D, et al. Exogenous Dp71 is a dominant negative competitor of dystrophin in skeletal muscle. Neuromuscul Disord. 2002;12:836-44.

S10. Lim KRQ, Shah MNA, Woo S, Wilton-Clark H, Zhabyeyev P, Wang F, et al. Natural History of a Mouse Model Overexpressing the Dp71 Dystrophin Isoform. Int J Mol Sci. 2021;22.

S11. Melacini P, Fanin M, Danieli GA, Villanova C, Martinello F, Miorin M, et al. Myocardial involvement is very frequent among patients affected with subclinical Becker's muscular dystrophy. Circulation. 1996;94:3168-75.

S12. Nicolas A, Raguenes-Nicol C, Ben Yaou R, Ameziane-Le Hir S, Cheron A, Vie V, et al. Becker muscular dystrophy severity is linked to the structure of dystrophin. Human molecular genetics. 2015;24:1267-79.

S13. Bello L, Gordish-Dressman H, Morgenroth LP, Henricson EK, Duong T, Hoffman EP, et al. Prednisone/prednisolone and deflazacort regimens in the CINRG Duchenne Natural History Study. Neurology. 2015;85:1048-55.

S14. Donovan J KN, Gordon G, Barthel B, DuVall M, Bronson A, Russell A, Sherman C, Evanchik M. P.124 EDG-5506 targets fast skeletal myosin and reduces muscle damage biomarkers in a phase 1 trial in Becker muscular dystrophy (BMD). Neuromuscular Disorders. 2022;32:S100.

**References Cited in Supporting Information text:**

1. Bittel AJ, Sreetama SC, Bittel DC, Horn A, Novak JS, Yokota T, et al. Membrane Repair Deficit in Facioscapulohumeral Muscular Dystrophy. Int J Mol Sci. 2020;21:doi:10.3390/ijms21155575

2. Petrof BJ, Shrager JB, Stedman HH, Kelly AM, Sweeney HL. Dystrophin protects the sarcolemma from stresses developed during muscle contraction. Proc Natl Acad Sci U S A. 1993;90:3710-4. doi:10.1073/pnas.90.8.3710

3. Heier CR, Yu Q, Fiorillo AA, Tully CB, Tucker A, Mazala DA, et al. Vamorolone targets dual nuclear receptors to treat inflammation and dystrophic cardiomyopathy. Life Sci Alliance. 2019;2:doi:10.26508/lsa.201800186

4. Coley WD, Bogdanik L, Vila MC, Yu Q, Van Der Meulen JH, Rayavarapu S, et al. Effect of genetic background on the dystrophic phenotype in mdx mice. Human molecular genetics. 2016;25:130-45. doi:10.1093/hmg/ddv460

5. Landini G, Martinelli G, Piccinini F. Colour deconvolution: stain unmixing in histological imaging. Bioinformatics. 2021;37:1485-7. doi:10.1093/bioinformatics/btaa847

6. Kinder TB, Heier CR, Tully CB, Van der Muelen JH, Hoffman EP, Nagaraju K, et al. Muscle Weakness in Myositis: MicroRNA-Mediated Dystrophin Reduction in a Myositis Mouse Model and Human Muscle Biopsies. Arthritis Rheumatol. 2020;72:1170-83. doi:10.1002/art.41215

7. Mayeuf-Louchart A, Hardy D, Thorel Q, Roux P, Gueniot L, Briand D, et al. MuscleJ: a high-content analysis method to study skeletal muscle with a new Fiji tool. Skelet Muscle. 2018;8:25. doi:10.1186/s13395-018-0171-0
